# Atomic Model of Rabbit Hemorrhagic Disease Virus by Cryo-Electron Microscopy and Crystallography

**DOI:** 10.1371/journal.ppat.1003132

**Published:** 2013-01-17

**Authors:** Xue Wang, Fengting Xu, Jiasen Liu, Bingquan Gao, Yanxin Liu, Yujia Zhai, Jun Ma, Kai Zhang, Timothy S. Baker, Klaus Schulten, Dong Zheng, Hai Pang, Fei Sun

**Affiliations:** 1 National Laboratory of Biomacromolecules, Institute of Biophysics (IBP), Chinese Academy of Sciences (CAS), Beijing, China; 2 University of Chinese Academy of Sciences, Beijing, China; 3 Department of Biochemistry and Molecular Biology, College of Life Sciences, Beijing Normal University, Beijing, China; 4 State Key Laboratory of Veterinary Biotechnology, Harbin Veterinary Research Institute, Chinese Academy of Agricultural Science, Harbin, China; 5 Beckman Institute and Department of Physics, University of Illinois at Urbana-Champaign, Urbana, Illinois, United States of America; 6 Department of Chemistry and Biochemistry and Division of Biological Sciences, University of California-San Diego, La Jolla, California, United States of America; 7 Analytical and Testing Center, Beijing Normal University, Beijing, China; 8 School of Medicine, Tsinghua University, Beijing, China; The Scripps Research Institute, United States of America

## Abstract

Rabbit hemorrhagic disease, first described in China in 1984, causes hemorrhagic necrosis of the liver. Its etiological agent, rabbit hemorrhagic disease virus (RHDV), belongs to the *Lagovirus* genus in the family *Caliciviridae*. The detailed molecular structure of any lagovirus capsid has yet to be determined. Here, we report a cryo-electron microscopic (cryoEM) reconstruction of wild-type RHDV at 6.5 Å resolution and the crystal structures of the shell (S) and protruding (P) domains of its major capsid protein, VP60, each at 2.0 Å resolution. From these data we built a complete atomic model of the RHDV capsid. VP60 has a conserved S domain and a specific P2 sub-domain that differs from those found in other caliciviruses. As seen in the shell portion of the RHDV cryoEM map, which was resolved to ∼5.5 Å, the N-terminal arm domain of VP60 folds back onto its cognate S domain. Sequence alignments of VP60 from six groups of RHDV isolates revealed seven regions of high variation that could be mapped onto the surface of the P2 sub-domain and suggested three putative pockets might be responsible for binding to histo-blood group antigens. A flexible loop in one of these regions was shown to interact with rabbit tissue cells and contains an important epitope for anti-RHDV antibody production. Our study provides a reliable, pseudo-atomic model of a *Lagovirus* and suggests a new candidate for an efficient vaccine that can be used to protect rabbits from RHDV infection.

## Author Summary

Rabbit hemorrhagic disease (RHD), first described in China in 1984, causes hemorrhagic necrosis of the liver within three days after infection and with a mortality rate that exceeds 90%. RHD has spread to large parts of the world and threatens the rabbit industry and related ecology. Its etiological agent, rabbit hemorrhagic disease virus (RHDV), belongs to the *Lagovirus* genus in the family *Caliciviridae*. Currently, the absence of a high-resolution model of any lagovirus impedes our understanding of its molecular interactions with hosts and successful design of an efficient anti-RHDV vaccine. Here, we use hybrid structural approaches to construct a pseudo-atomic model of RHDV that reveals significant differences in the P2 sub-domain of the major capsid protein compared to that seen in other caliciviruses. We identified seven regions of high sequence variation in this sub-domain that dictate the binding specificities of histo-blood group antigens. In one of these regions, we identified an antigenic peptide that interacts with rabbit tissue cells and elicits a significant immune response in rabbits and, hence, protects them from RHDV infection. Our pseudo-atomic model provides a structural framework for developing new anti-RHDV vaccines and will also help guide use of the RHDV capsid as a vehicle to display human tumor antigens as part of anti-tumor therapy.

## Introduction

Rabbit hemorrhagic disease (RHD) is extremely contagious in adult rabbits and is often associated with liver necrosis, hemorrhaging, and high mortality [1]. It was first described in China in 1984 [2], and within a few years had spread worldwide [3]. RHD outbreaks still occur on almost every continent and cause significant mortality rates, being endemic in Europe, Asia, Africa, and Australia [4]. This disease has a significant impact on the rabbit industry and ecology [4].

The etiological agent of RHD is rabbit hemorrhagic disease virus (RHDV), which has a single-stranded, positive-sense, polyadenylated RNA genome of ∼7.5 kb [5]. Mature RHDV virions are spherical, non-enveloped particles with a T = 3, icosahedral capsid whose outer diameter varies between 32 and 44 nm and whose structure is defined by characteristic, cup-shaped depressions [6]. The only capsid protein present in RHDV, VP60, is composed of three domains, which include the N-terminal arm (NTA), the shell (S), and the protrusion (P), the latter of which is further divided into P1 and P2 sub-domains [7].

RHDV belongs to the genus *Lagovirus* of the family *Caliciviridae*, which also includes the genera *Norovirus*, *Nebovirus*, *Sapovirus* and *Vesivirus* [8], [9]. Previous structural studies of caliciviruses include three-dimensional (3D) cryo-electron microscopic (cryoEM) reconstructions of virus-like particles (VLPs) of Murine Norovirus (MNV, *Norovirus*) and Feline calicivirus (FCV, *Vesivirus*) at 8- and 16-Å resolution, respectively [10], [11], and determination of the crystal structures of the Norwalk virus (NV, *Norovirus*) capsid at 3.4 Å [12], native FCV virions at 3.6 Å [13], and native virions of San Miguel sea lion virus (SMSV, *Vesivirus*) at 3.2 Å [14]. CryoEM reconstructions of the RHDV VLP at 8 Å [15] and the native RHDV virion at 11 Å [7] have been computed and a C_α_ homology model of RHDV was built based on the VLP cryo-reconstruction by using the crystal structure models of SMSV and FCV [16]. However, a more complete atomic model of RHDV is still lacking. Furthermore, the P domain of VP60, which is responsible for antigenicity and binding to host tissue [17], varies considerably across different *Caliciviridae* species, and hence this stimulated us to crystallize and obtain a high resolution crystal structure of this domain to provide a model that is more reliable than could be gleaned from any homology modeling approach.

It is worth noting that noroviruses infect hosts by recognizing histo-blood group antigens (HBGAs) that are important host susceptibility factors [18], and RHDV also agglutinates human erythrocytes and attaches to epithelial cells in the upper respiratory and digestive tracts of rabbits by binding to HBGAs [19]. HBGAs have recently been shown to act as attachment factors that facilitate infection and RHDV isolates from six different genetic groups bind specifically to different HBGAs [20].

Here, we report a pseudo-atomic model of the RHDV capsid derived through a combination of X-ray crystallography, cryoEM reconstruction, and molecular dynamics flexible-fitting (MDFF) [21]. We find that RHDV VP60 has a P2 sub-domain that differs from other caliciviruses. Furthermore, our new model reveals that certain aspects of the P2 and NTA domain structures that were previously reported [16] need reinterpretation. We also examined the putative HBGA binding sites in RHDV by mapping isolate–related sequence variations onto the P domain structure. Finally, we show that a peptide derived from a putative HBGA binding site can interact with hosts and stimulate the production of virus antibody. The new, high-resolution model of a *Lagovirus* presented here provides a solid framework for developing an efficacious antigen presenting system. The model yields also new insights regarding the molecular mechanisms of RHDV-host interactions.

## Results/Discussion

### CryoEM reconstruction of the RHDV virion

**Figure 1:**
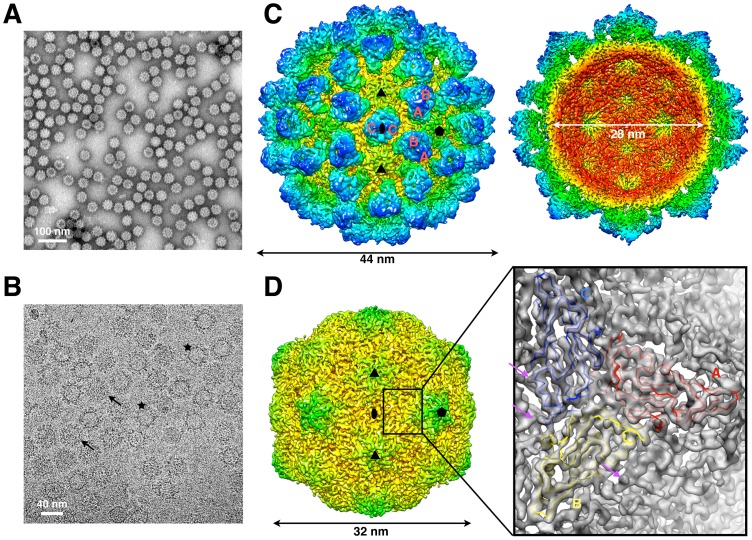
Electron microscopy and 3D image reconstruction of RHDV. (**A**) Micrograph of purified, negatively stained RHDV (bar = 100 nm). (**B**) CryoEM micrograph of purified RHDV (bar = 40 nm). The black arrows and stars point to RHDV particles with differing amounts of internal density (also see **Figure S1**). (**C**) Reconstructed cryoEM map of the RHDV virion, color-coded by radius. Icosahedral 2-, 3- and 5-fold axes are indicated by black symbols and AB and CC capsomers are identified. On the right, the closest half of the density map has been removed to reveal internal features in the RHDV density map. The contour threshold of the cryoEM map here was set to 3.3σ above the mean. (**D**) Same as (C) but with the capsomer density removed to show just the RHDV inner shell. Insert: A region of the density map is shown as a transparent grey isosurface into which is fitted the backbone structures of the S domains from three copies of the RHDV major capsid protein, VP60 (A in red, B in yellow and C in blue). Magenta arrows point to a few representative density features that represent residue side chains.

Highly purified RHDV virions (**Figure 1A**) obtained from the livers of infected domestic rabbits were used for crystallization trials and cryoEM studies (**Figure 1B**). Unfortunately, we were unable to obtain any crystals of RHDV suitable for X-ray diffraction owing to its propensity to degrade with time. From cryoEM micrographs (**Figure 1B**), consistent with previous observations [7], [22], two distinct classes of particles were observed: intact virions containing whole genomic RNA (high density inside) and “empty” virions containing sub-genomic RNA (low density inside). The presence of these two types of particles was confirmed by image classification (**Figure S1A**). The cryoEM structure of RHDV that we computed from ∼36,000 images of individual particles (**Figure 1C** **and S1B**) was estimated to reach a resolution limit of 6.5/4.8 Å (**Figure S1C**) based on Fourier shell correlation (FSC) cutoff thresholds of 0.5 and 0.143, respectively [23], [24]. Considerably more detail was resolved in this RHDV cryo-reconstruction compared to that in our previous one at 11 Å [7]. In addition, the resolution achieved in the RHDV inner shell (radii between ∼130 and 150 Å) reached 5.5 Å (FSC_0.5_; **Figure S1C**) compared to 7.0 Å (FSC_0.5_) for structural features at larger radii (between ∼150 and 220 Å). Central cross sections of the reconstructed 3D map taken perpendicular to the icosahedral 3-, 5-, and 2-fold axes show well-resolved densities in the inner shell compared to fuzzier densities at larger radii (**Figure S1B**), consistent with the protruding capsomers exhibiting high flexibility [7], [15]. All secondary structural elements in the VP60 S domain were clearly resolved and, in some regions, densities corresponding to residue side chains were evident (**Figure 1D**). Compared to reconstructions of the RHDV VLP at 8 Å [15] and the native virion at 11 Å [7], the present result represents the most detailed view of the RHDV capsid structure and this, along with results from X-ray crystallography, enabled us to build a reliable, pseudo-atomic model.

As shown previously [7], the RHDV capsid has an overall spherical shape, with a maximum outer diameter of 44 nm and an inner chamber with a diameter of 28 nm (**Figure 1C**). The asymmetric unit of the RHDV capsid consists of three, quasi-equivalent VP60 subunits (A, B and C) arranged with T = 3 icosahedral symmetry. The 180 VP60 subunits that comprise the capsid are organized as 90 dimers, each of which appears as an arch-like capsomer. Thirty C/C capsomers are located at the icosahedral two-fold symmetry axes and the remaining 60 A/B capsomers are located at pseudo (“local”) two-fold axes. Three A/B and three C/C dimers are positioned in alternate fashion around each icosahedral three-fold axis to form pseudo-six-fold arrangements, and five A/B dimers encircle each five-fold axis. Together, these capsomers produce a contiguous shell and 32 cup-shaped, surface depressions, the latter of which are a characteristic feature of the structure of all caliciviruses [25].

### Crystal structures of the VP60 S and P domains

**Figure 2:**
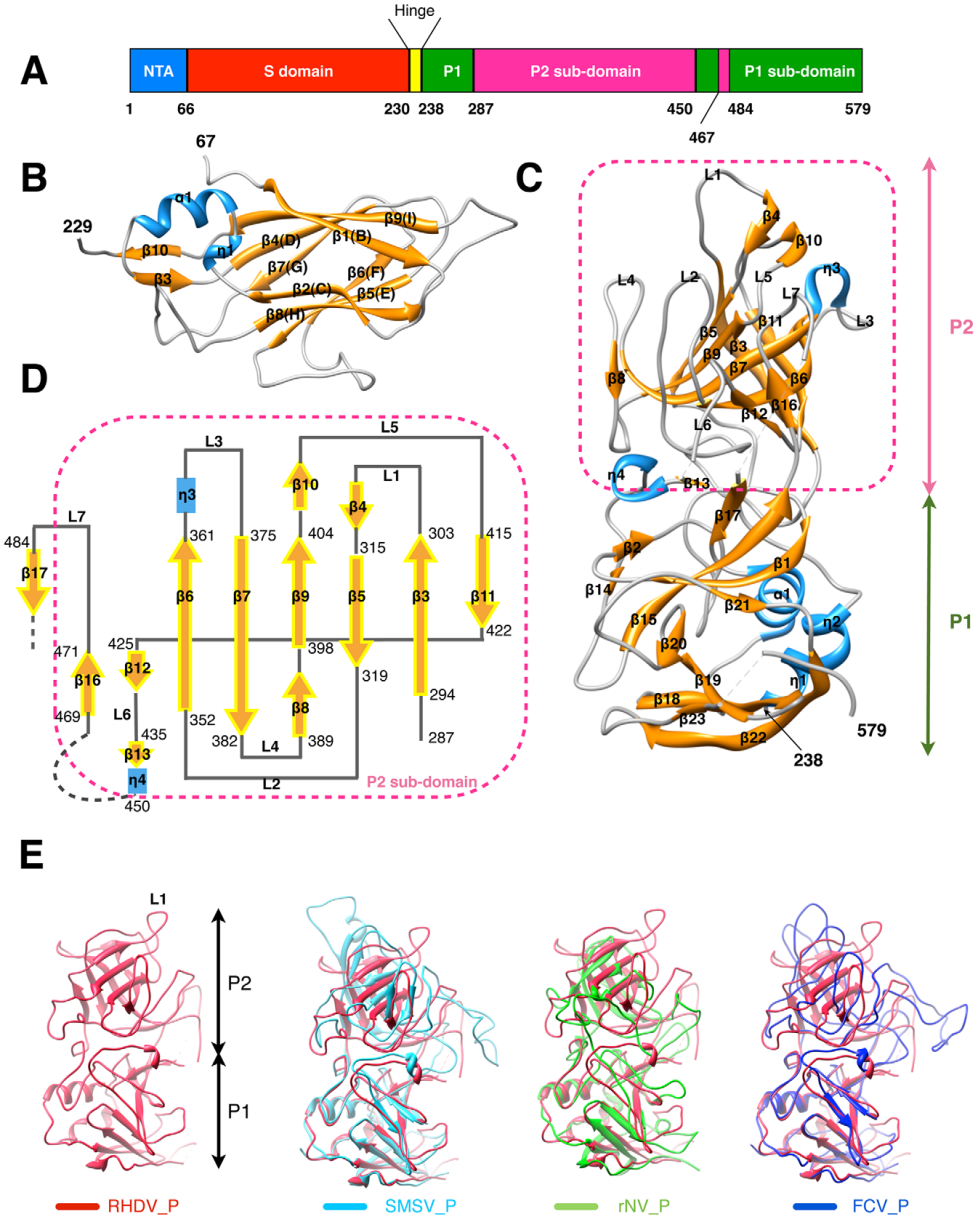
Crystal structures of the S and P domains of RHDV major capsid protein VP60. (**A**) Domain organization of VP60. (**B**) Ribbon representation of the crystal structure of the VP60 S domain showing the classic viral, BIDG-CHEF β-barrel motif. Secondary structures are colored blue for helices, gold for β-strands, and grey for loops and are labeled sequentially (see also **Figure S2**). (**C**) Ribbon representation of the crystal structure of the VP60 P domain. P1 (green) and P2 (pink) sub-domains are indicated and colored according to their secondary structure elements and labeled sequentially (see also **Figure S4**). (**D**) Topology diagram of the VP60 P2 sub-domain. Labels and residue numbers correspond to those shown in panel (**C**). (**E**) Crystal structure of the VP60 P domain of RHDV (red) is superimposed with the crystal structures of the VP60 P domains in rNV (green), SMSV (cyan), and FCV (blue). The L1 loop of the RHDV VP60 P domain is labeled.

RHDV VP60 is subdivided into three domains, NTA (the N-terminal arm, a.a. 1–65), S (the shell, a.a. 66–229), P (the protrusion, a.a. 238–579) and a short hinge (a.a. 230–237) that connects S and P (**Figure 2A**). The S domain together with the NTA domain (a.a. 1–230) was cloned and expressed in *E.coli*, purified, and crystallized in space group C2. We solved the crystal structure of the S domain by molecular replacement and refined it to a resolution limit of 2.0 Å with final R_work_ and R_free_ values of 20.0% and 24.1%, respectively (**Table 1**). The NTA domain could not be traced owing to lack of electron density, though SDS-PAGE analysis of crystals did not exhibit any obvious protein degradation. This indicates that the NTA domain is inherently quite flexible in crystals. The S domain of RHDV shares high sequence homology with the S domains of other caliciviruses (**Figure S2A**) and folds into a canonical, eight-stranded, BIDG-CHEF β-barrel [26] (**Figure 2B**). The structure of the RHDV S domain superimposes quite closely with the corresponding S domains of FCV, SMSV, and NV (**Figure S2B**). The root mean squared deviations (r.m.s.d) of the C_α_ coordinates of the RHDV S domain compared to each of these three viruses are 1.51 Å (149 Cα), 1.41 Å (150 Cα), and 1.32 Å (153 Cα), respectively, suggesting that the structures of the inner shells of all caliciviruses are highly conserved.

**Table 1:** Statistics for crystallographic data collection and processing.

Sample	P-domain	S-domain
**Data collection**		
Space group	P2_1_2_1_2_1_	C2
Cell Dimensions		
a, b, c (Å)	43.9, 88.1, 135.6	125.7, 48.4, 65.2
α, β, γ (°)	90.0, 90.0, 90.0	90.0, 101.0, 90.0
Resolution (Å)	50.00–2.00 (2.03–2.00)	50.00–2.00 (2.07–2.00)
R^a^_merge_	0.089 (0.231)	0.086 (0.404)
I/σ	29.8 (8.3)	25.7 (4.0)
Completeness (%)	99.1 (99.9)	100.0 (99.8)
Redundancy	3.7 (3.9)	7.4 (6.8)
**Refinement**		
Resolution (Å)	50.00–2.00	50.00–2.00
No. unique reflections	33,925	24,815
R_work_/R_free_ (%)^b^	19.9/23.1	20.0/24.1
r.m.s.d. bond lengths (Å)	0.009	0.027
r.m.s.d. bond angles (°)	1.248	2.109

Corresponding parameters for the highest resolution shell are shown in parentheses.

a R_merge_ = Σ_h_Σ_i_|*I*_ih_−<*I*_h_>|/Σ_h_Σ_i_<*I*_h_>, where <*I*_h_> is the mean intensity of the observation *I*_ih_ reflection h.

b R_work_ = Σ(∥F_p_(obs) |−|F_p_(calc) ∥)/Σ|F_p_(obs) |; R_free_ = R factor for a selected subset (5%) of the reflections that was not included in prior refinement calculations.

The fragment (a.a. 228–579) that includes the entire VP60 P domain was expressed in a baculovirus system, purified, and formed crystals that belong to space group P2_1_2_1_2_1_. Its crystal structure (**Figure 2C**) was determined by molecular replacement, with the capsomer portion of the RHDV cryoEM density map used for initial phasing. This structure was refined to a resolution of 2.0 Å with final R_work_ and R_free_ values of 19.9% and 23.2%, respectively (**Table 1** **and Figure S3**). The asymmetric unit of the crystal contains a dimer of P domains. The P domain of RHDV, like in other caliciviruses [12], consists of sub-domains P1 (a.a. 238–286, 450–466, 484–579) and P2 (a.a. 287–449 and 467–483) (**Figure 2A, C and D**). The P1 sub-domain of RHDV has a conserved fold compared to caliciviruses FCV, SMSV, and NV, with r.m.s.d values for the C_α_ coordinates of 1.53 Å (144 Cα), 1.49 Å (145 Cα), and 2.14 Å (134 Cα), respectively (**Figure 2E**). The P2 sub-domain has a predominant β-barrel core comprised of six anti-parallel β strands (β6-β7-β9-β5-β3-β11) folded in a Greek-key topology and a two-stranded β sheet (β12–β16), which are connected by seven loops (L1–L7) of various lengths and surrounded by two short helices (η3 and η4) (**Figure 2C and D**). The P2 sub-domains of RHDV, NV, SMSV, and FCV exhibit no obvious sequence homology (**Figure S4**), and the C_α_ coordinate r.m.s.d between the P2 sub-domain of RHDV and that of NV, SMSV, and FCV are 3.00 Å (38 C_α_), 2.68 Å (123 C_α_), and 4.32 Å (107 C_α_), respectively. Although they share a consensus β-barrel core, the loop regions differ significantly (**Figure 2E**) and are expected to be a primary determinant of the host range for each particular virus.

### Atomic model of the complete RHDV capsid

**Figure 3:**
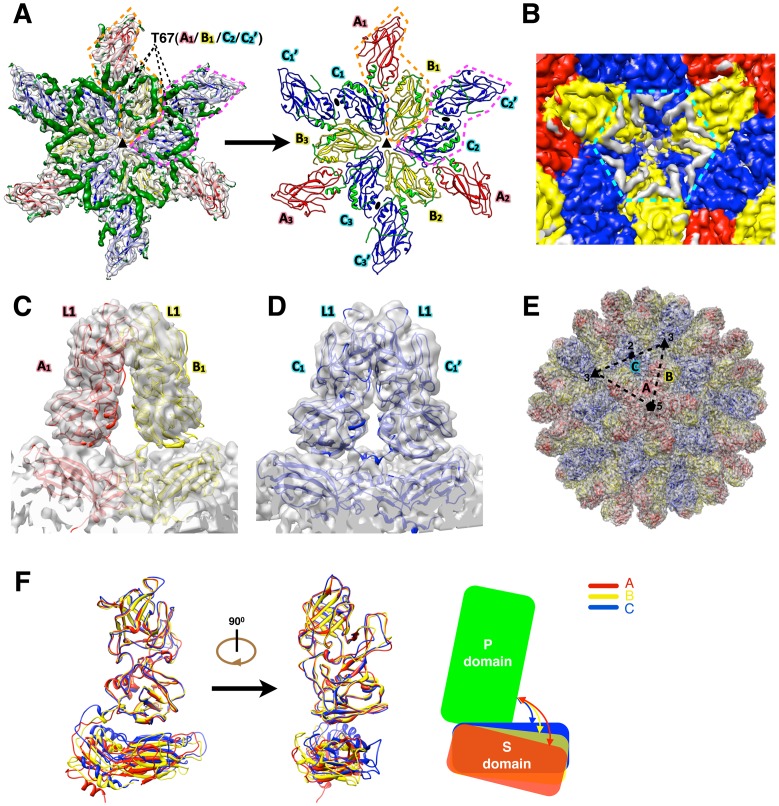
Atomic model of RHDV capsid. (**A**) Modeling the NTA domain. The crystal structure of the VP60 S domain was fitted to and refined against the cryoEM map of RHDV. The S domains from quasi-equivalent VP60 monomers A, B and C, are colored red, yellow, and blue, respectively. Dashed arrows point to the N-terminal, T67 residues resolved in the crystal structures. A/B and C/C dimers are highlighted by dashed orange and pink polygons, respectively. Difference densities between the fitted models and cryoEM map, shown in dark-green around a 3-fold axis, were used to trace and build the NTA segments of VP60. The final pseudo-atomic model of the RHDV inner shell, including the NTA segments (green) around the 3-fold axis, is shown in ribbon form on the right. (**B**) CryoEM density reveals that the NTA segments of the B and C monomers extend to the proximal 3-fold axis and interact with each other. Densities corresponding to the A, B, and C monomers are colored red, yellow, and blue, respectively and those for the NTA domains are colored grey and are surrounded by a dashed cyan hexagon. (**C**, **D, E**) The match between the final model and the cryoEM map for the A/B capsomer in (**C**), for the C/C capsomer in (**D**) and for the whole virus in (**E**). The color scheme is that used in (**A**). In (**C**) and (**D**), the L1 loop at the top surface of capsomers A/B and C/C is labeled. In (**E**), the whole pseudo-atomic model of RHDV is shown in ribbon representation and fitted into the cryoEM map and viewed along a 5-fold axis. Black triangles and an ellipse mark the positions of two 3-fold axes and a single 2-fold axis, respectively. (**F**) Atomic models of three quasi-equivalent RHDV VP60 monomers, A (red), B (yellow), and C (blue). The three models, represented in ribbon form, are superimposed with the P domains aligned. Conformational differences among the three subunits are shown in schematic form at the right, which highlights alternative orientations that the S domains adopt relative to P.

The crystal structures of the S and P domains of VP60 were docked into the high-resolution cryoEM map to construct a pseudo-atomic model of the complete RHDV capsid. With the exception of a few loops, the S domain fit quite well into the density map (**Figure 3A**). Despite the absence of density for the NTA domain in the crystal structure of the NTA-S recombinant molecule (**Figure 2B**), a difference map computed by subtracting the fitted S domain model from the RHDV virion cryoEM map enabled us to build an *ab initio* model of the NTA domain (residues 30–65) (**Figure 3A**). At each three-fold axis of the virion, three A/B and three C/C dimers pack in alternate fashion via their S domains and clear densities at the interface of each dimer show that each NTA domain folds onto its cognate S domain (**Figure 3A** **, S5A and B**). The NTA domains of the B and C monomers form a network of interactions with a plug-like density (formed by residues 1–30) surrounding the three-fold axis (**Figure 3A and B**) as was also described previously [16]. Contacts formed by the NTA domains in the inner shell of the virion confirm the importance of this domain for virion assembly, which concurs with previous truncation [27] and insertion studies [16].

The folding back of NTA onto the S domain of the same VP60 subunit in RHDV is similar to that seen in NV [12], but differs from that in SMSV, where the NTA domain extends away from the cognate S domain to interact only with the S domain in an adjacent subunit [14]. The cryoEM density map of RHDV showed that the protruding regions of the A/B and C/C dimers only interact between the P2 sub-domains (**Figure 3C and D**), which is consistent with the crystal structure of SMSV [14]. However, the NV crystal structure shows that these dimers include P1-P1 as well as P2-P2 interactions [12].

Following initial rigid-body docking of the crystal structures of the S and P domains into the RHDV cryoEM map along with the modeled NTA segments, MDFF procedures [21] were used to build a complete, pseudo-atomic model of the capsid (**Figure 3E**). The refined model fits the cryoEM map very well for both P and S domains with apparently good consistence (**Figure 3C and D** **, S5C and D**). Furthermore, comparison of the MDFF-refined model with the initial rigid-body-fit model, showed that the local cross correlation coefficient between the atomic model and the cryoEM map improved from 0.473 to 0.634 (**Table S1**). The r.m.s.d between the initial model and MDFF-refined model is 2.45 Å. In particular, the local cross correlation coefficient for the S domain improved from 0.452 (before MDFF) to 0.673 (after MDFF). MDFF not only improved the fitting in the loop region around the 3-fold axis, but also closed the gaps between B and C subunits at the interface (**Figure S5E and F**). The improvements in local cross correlation coefficients for other domains are given in **Table S1**. Structural comparisons among the A, B and C monomers of the MDFF-refined model, when aligned to the P domains, revealed that large conformational changes accompany relative movements and rotations of the S domain with respect to the P domain (**Figure 3F**). The complete, pseudo-atomic model of the RHDV capsid exhibits the classic calicivirus features: an inner shell formed by 180 S domains and 90 protrusions formed by dimeric arrangements of the P domains (**Figure 3E** **and** **Movie S1**).

Next, we compared our current structural model of RHDV with the previously reported backbone model derived from the 8.0 Å VLP cryo-reconstruction and homology modeling [16]. Comparison of the three quasi-equivalent monomers (A, B and C) in the two models (**Figure S6**) showed that the relative positions of the P and S domains correspond closely to each other, but that nevertheless two significant differences are found. First, the NTA domain in the previous model extends to interact with the S domain in an adjacent subunit, whereas the current model shows instead that the NTA domain folds back onto its cognate S domain. Second, except for the β-barrel core motif, the loop regions in the P2 sub-domains differ completely between the two models. As a result, our higher resolution cryoEM map of the RHDV capsid (especially in the inner shell) and the crystal structure of the P domain together provide more accurate structural details about the NTA domain and P2 sub-domain. This detail lays a foundation for understanding how RHDV interacts with its hosts and how the virus displays a specific antigenic epitope.

### Variation of RHDV capsomer outer surface and the putative HBGA binding sites

**Figure 4:**
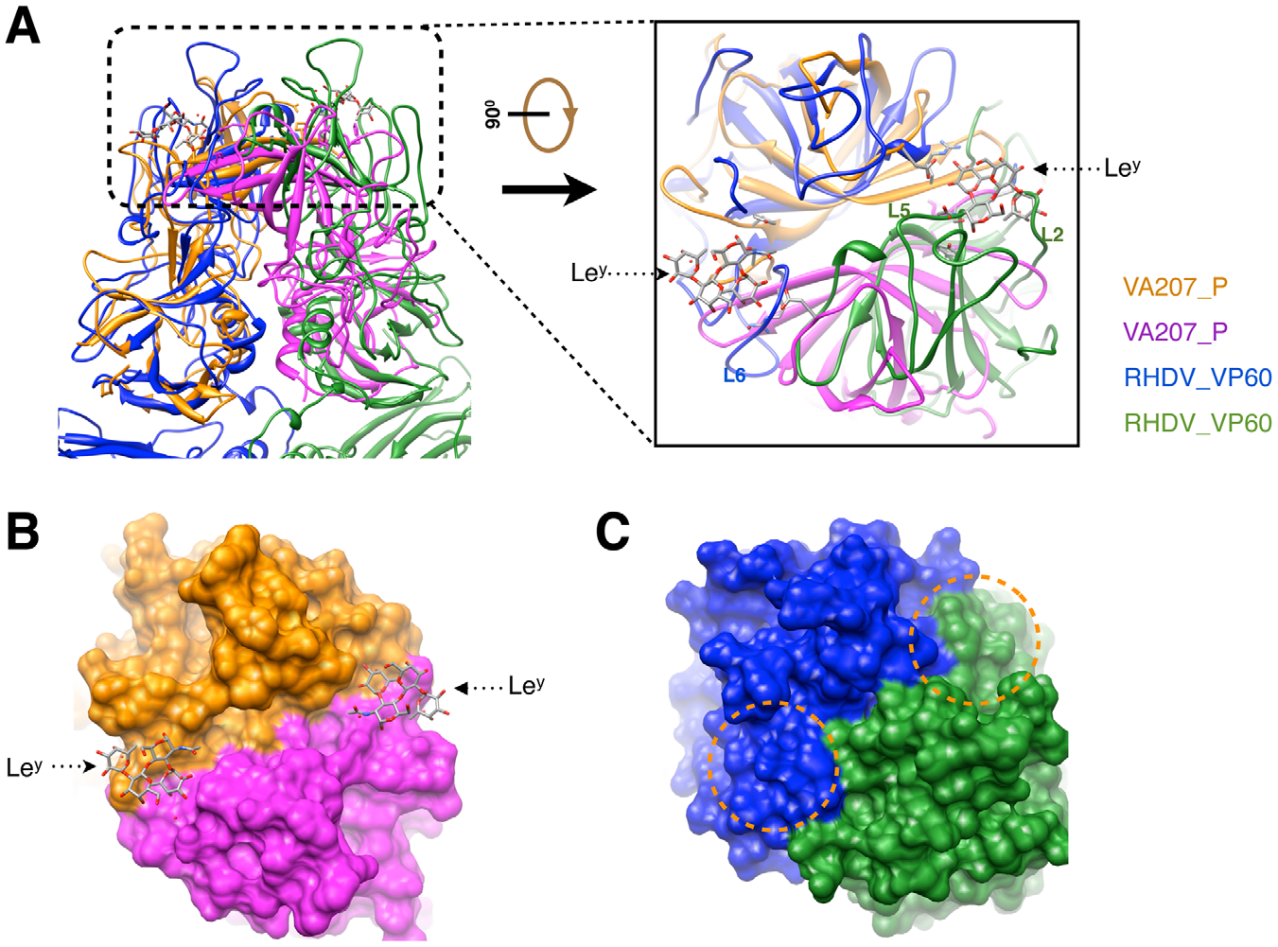
Structural comparison of histo-blood group antigens (HBGAs) binding sites in RHDV and NV VP60s. (**A**) RHDV C/C capsomer (blue and green) is superimposed with the crystal structure of the norovirus strain (VA207, GII.9) [18] (PDB code: 3PUN) P dimer (gold and magenta). A close-up, top-down view of the HBGAs binding sites in VA207 is shown at the right and compared with the RHDV P2 sub-domains. Le^y^ represents the HBGAs Lewis y. (**B**, **C**) Surface representations of the dimeric P domains in NV VA207 (**B**) and RHDV (**C**) are shown in equivalent top views and colored as in (**A, right panel**). The portions of RHDV that correspond to the Le^y^ binding sites in NV VA207 are indicated with orange dashed circles in (**C**).

The first step of viral entry in NV and RHDV infections involves recognition of HBGAs [18], [19]. Crystal structures of NV variants V387 and V207, bound with HBGAs, revealed that the binding sites in NV are located at the outer surface of the arch-like P dimers with both P domains contributing to the formation of the binding interfaces [18], [28]. Because the structure of the RHDV P domain bound with HBGAs is currently not available, we selected the crystal structure of NV variant V207 complexed with the non-secretor HBGA Lewis y (Le^y^) tetrasaccharide as a model (PDB code 3PUN) [18] to compare with our atomic model of RHDV VP60 (**Figure 4A**). The crystal structure of the NV V207 P dimer was superimposed onto the C/C capsomer of RHDV by aligning to one of the subunits. The relative positions of the two subunits within the dimer differ slightly between the NV V207 and RHDV models. The binding site of the Le^y^ tetrasaccharide in the P dimer of NV V207 corresponds to loop L6 or L2 in the P domain of RHDV VP60 (**Figure 4A**). A surface representation of the NV P dimer shows that Le^y^ tetrasaccharides bind to the outer portion of the dimeric interface between P domains (**Figure 4B**). However, this interface is completely different in RHDV (**Figure 4C**), and therefore, the RHDV capsomer likely utilizes distinct binding sites for HBGAs.

**Figure 5:**
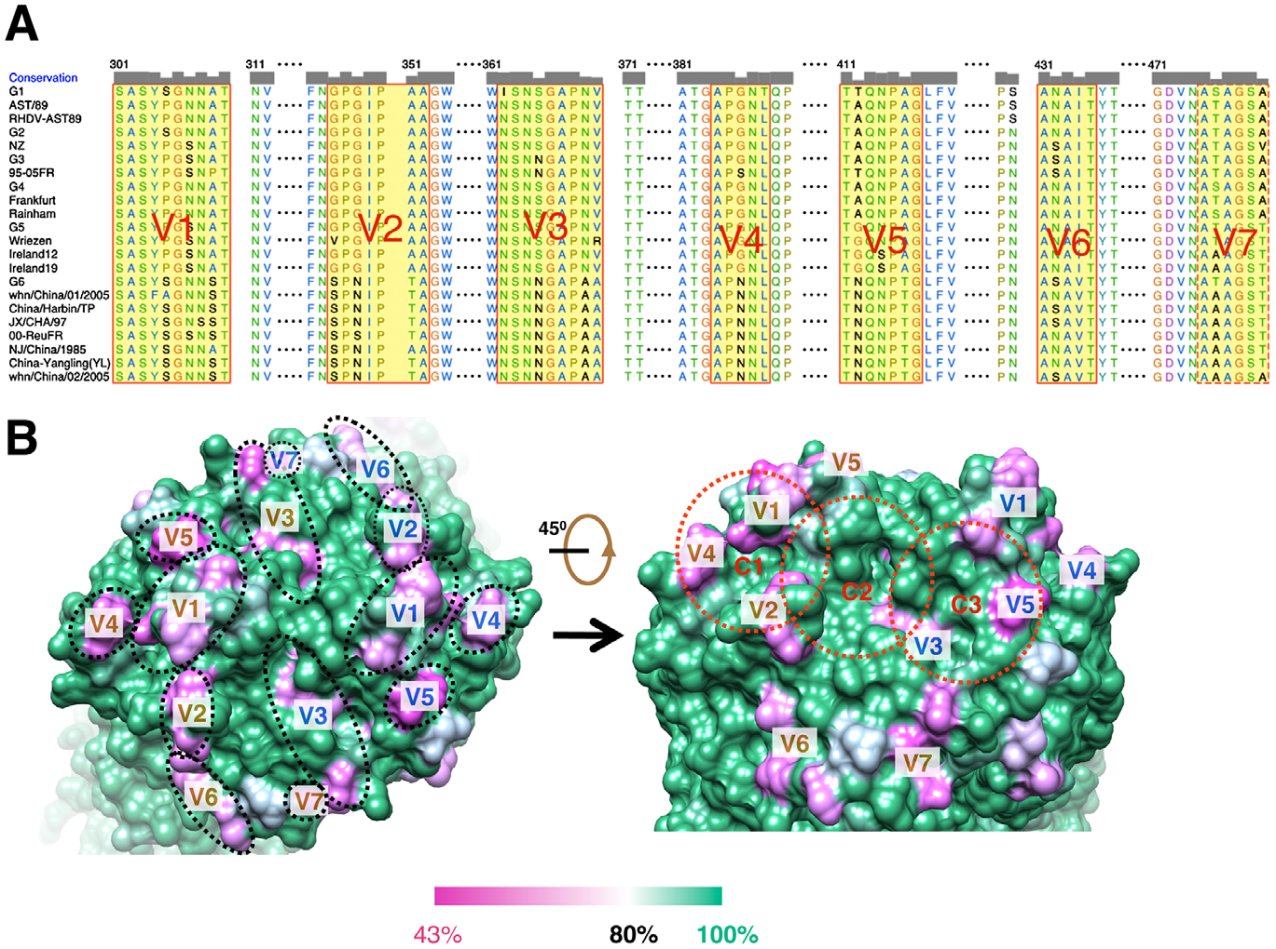
Sequence alignment of VP60s of representative RHDV isolates and location of variation regions on the capsomer surface. (**A**) Multiple sequence alignments of VP60s among six genetic groups of RHDV isolates according to Nystrom, K. et.al. [20]. The alignment is just shown for the P2 sub-domain region for residues from 301 to 480. The GeneBank accession numbers for those isolates shown are JF438967 (G1), Z24757 (AST/89), Z49271 (RHDV-AST89), FR823355 (G2), AF231353 (NZ), FR823354 (G3), AJ535092 (95-05FR), AJ535094 (G4), Y15424 (Frankfurt), AJ006019 (Rainham), AM085133 (G5), Y15427 (Wriezen), AY926883 (Ireland12), AY928269 (Ireland19), AJ969628 (G6), DQ069280 (whn/China/01/2005), AF453761 (China/Harbin/TP), DQ205345 (JX/CHA/97), AJ303106 (00-ReuFR), AY269825 (NJ/China/1985), DQ530363 (China-Yingling(YL)), and DQ069281 (whn/China/02/2005). The seven variation regions (V1–V7) that distinguish these isolates are highlighted in yellow. Sequence alignments were performed and plotted using Chimera [39]. (**B**) Locations of variation regions on the RHDV capsomer surface. The degrees of sequence conservation based on the multiple sequence alignments in (**A**) are mapped onto the surface of the RHDV capsomer (shown in top view on the left). The most highly conserved regions (between 80 and 100% conservation) are shown in colors varying from white to cyan and then green, whereas less conserved regions (from 80 down to 43%) are represented in shades from white to violet. The seven variation regions in each of the two P2 sub-domains are indicated by dashed black ellipses and are labeled in gold in one monomer and blue in the other. The right panel shows a view of the variation sites from a different angle and three putative binding sites for HBGAs are highlighted with dashed red circles and labeled C1, C2 and C3.

Though genetic diversity among RHDV isolates is far lower than that among isolates of other caliciviruses, it has been suggested that all current RHDV isolates could be assigned to one of six genetic groups and the binding specificities of HBGAs for those genetic groups have been the subject of intensive investigation recently [20]. We performed a multi-sequence alignment of VP60 among these six groups and found that seven regions of high variation (V1 to V7) distinguish these groups (**Figure 5A**). These regions all occur on the P2 sub-domain (**Figure 5B** **and S7**). Most significantly, these regions correspond to loops L1 to L7 in the P2 sub-domain (**Figure 2** **and** **5**). Thus, in addition to the antigenic variation contributed by these loop regions, at least some and perhaps all of these loops may give rise to different HBGA binding specificities. A relationship between variation regions and receptor binding specificity was also gleaned from the cryoEM structure of FCV bound with its receptor, fJAM-1 (feline junctional adhesion molecule 1) [11]. In addition, we found three cavities on the outer surface of the RHDV capsomer (labeled C1, C2 and C3 in **Figure 5B**), one or more of which might contribute to HBGA binding. Whether these are true binding sites awaits investigation by mutagenesis experiments.

### RHDV variation region V1 (loop L1) contributes to host interaction and is a major neutralization site

Variation region V1 is a contiguous stretch of mostly hydrophilic residues on loop L1 (a.a. 304–314) (**Figure 5A**) and is highly flexible in crystals as evidenced by high crystallographic B-factors (**Figure S8A and B**). Given that L1 is the most exposed loop on the surface of the RHDV capsomer and that it lies juxtaposed to three putative HBGA binding pockets (**Figure 5B**), this loop is hypothesized to be a primary determinant of RHDV host interaction such that it represents an effective epitope in RHDV. Also, the sequence of this loop constitutes the most diverse region in VP60 in RHDV isolates (**Figure S8C**) and suggests that this sequence plays a critical role in defining RHDV antigenicity.

**Figure 6:**
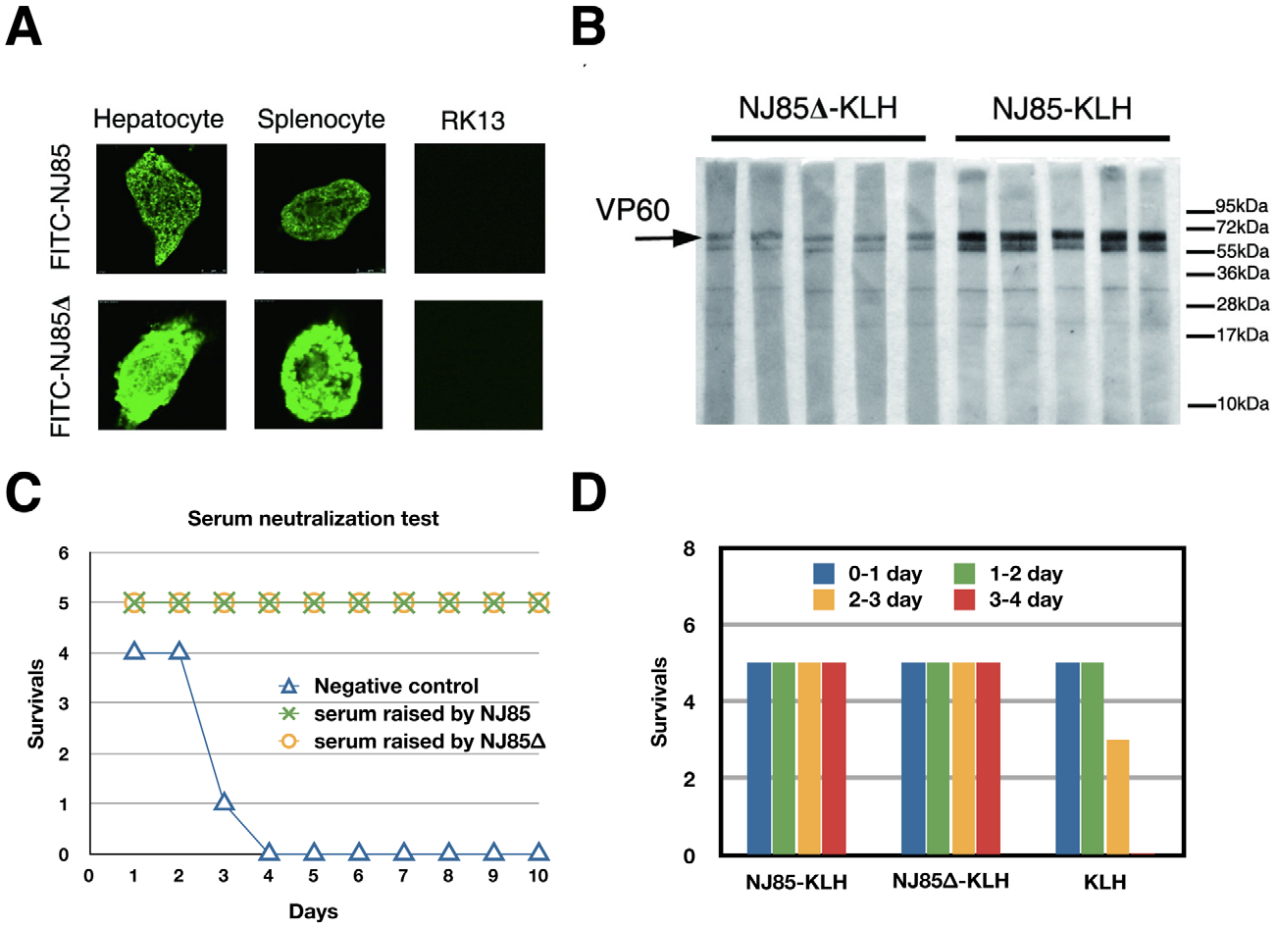
Variation region V1 of RHDV VP60. (**A**) Binding assay of three types of rabbit tissue cells (hepatocytes, splenocytes, and RK13 cells) for peptides derived from loop L1. (**B**) Western blot assay against RHDV for the sera containing anti-NJ85-KLH and anti-NJ85Δ-KLH antibodies. The black arrow indicates the band of the major capsid protein VP60 (see also **Figure S9**). (**C**) Virus neutralization assays for the sera raised against the NJ85 and NJ85Δ peptides. Rabbits were challenged by an RHDV sample that was mixed with the respective sera and monitored for the number of animals that survived as a function of time. (**D**) Efficiency of KLH-conjugated peptides (NJ85-KLH and NJ85Δ-KLH) in developing vaccines. Rabbits immunized with NJ85-KLH, NJ85Δ-KLH and KLH (control), were subjected to challenge against the HYD strain of RHDV. Each experimental group consisted of five rabbits and survival was monitored every day.

To test our hypothesis, we designed two peptides, NJ85 (a.a. 300–318) and NJ85Δ (missing 4 residues N_308_ATN_311_ of the loop L1 on the most exposed position of the capsomer), derived from the VP60 protein of the RHDV NJ85 isolate strain. Each peptide was synthesized with an N-terminally-labeled fluorescent isothiocyanate (FITC) and then used as a reagent to analyze receptor-binding activity in rabbit hepatocytes, primary splenocytes, and kidney (RK13) cells from healthy male, New Zealand white rabbits. Both peptides bound to the surfaces of the hepatocytes and primary splenocytes, but neither one bound to RK13 cells (**Figure 6A**). This suggests that the hepatocyte and splenocyte cells express receptors capable of binding both peptides, but that rabbit kidney cells do not, which concurs with previous studies on the specific tissue distributions of RHDV [29], [30]. This binding assay also suggested that at least one of the RHDV-host interaction sites resides at the top surface of the capsomer (*i.e.,* in the loop). However, the four residues (308–311) at the top part of the capsomer, which vary the most across isolates (**Figure S8C**), unexpectedly did not affect interactions between RHDV and its host.

To explore whether the protruding loop L1 of the capsomer can function as an antigenic site and induce an effective host immune response, we coupled the NJ85 and NJ85Δ peptides with keyhole limpet hemocyanin (KLH) (KLH-NJ85 and KLH-NJ85Δ) and used these two constructs to immunize rabbits. Western blot and ELISA analyses showed that antibody titer induced by KLH-NJ85 is about ten-fold higher than that induced by KLH-NJ85Δ (**Figure 6B** **and S9**). The RHDV hemagglutination inhibition assay revealed that the inhibition titers of the serum raised by those two peptides were 1∶64 for KLH-NJ85 and 1∶32 for KLH-NJ85Δ, respectively (**Figure S10**). As a result, although the highly exposed four residues (a.a.308–311) at the top of the capsomer are not required for host cell interaction, they do elicit a strong immunological response from the host.

We next investigated whether antibodies raised by those two peptides could neutralize RHDV and protect rabbits. Fifteen rabbits were divided into three groups of five. The sera that were raised by KLH-NJ85 and KLH-NJ85Δ were diluted 32-fold. An aliquot of each (800 µL) was mixed with 256 hemagglutination units of RHDV and incubated at 37°C for 1 hour, and then the mixture was given to rabbits intranasally. The serum from specific pathogen free rabbits was used as a negative control. In the negative control group, one of the five rabbits died within 24 hours after inoculation, three of the five rabbits died within 72 hours, and the fifth succumbed within 96 hours. In the groups of rabbits inoculated with sera raised by KLH-NJ85 and KLH-NJ85Δ, the rabbits were continuously housed and monitored every 24 hours for 10 days and all ten rabbits survived (**Figure 6C** **and Table S2**).

Furthermore, virus challenge with RHDV displayed 100% immune protection in the two groups of rabbits vaccinated separately with KLH-NJ85 and KLH-NJ85Δ (**Figure 6D**). In a control group that was vaccinated in parallel with KLH, two of the five rabbits died within 48 hours after challenge whereas the other three succumbed within 72 hours. Each virus challenge experiment was repeated four times and yielded consensus results (**Table S3**). These experiments demonstrated that loop L1 in the P2 sub-domain of VP60 forms an epitope on RHDV, and peptides derived from this loop are sufficient to stimulate rabbits to produce antibodies that immunize them against RHDV infection.

It is noteworthy that previous structural studies of a norovirus/Fab complex suggested the two loops (A′-B′ and E′-F′) in the P2 sub-domain of MNV to contact antibody [10], [31]. When we superimposed the crystal structures of the RHDV and MNV P2 sub-domains, we found that the L1 and L5 loops of RHDV correspond, respectively, to the two loops in MNV (**Figure S11**). Consequently, our results with RHDV concur with those of at least one other calicivirus.

It is unclear why peptides NJ85Δ and NJ85 provide equal protection from RHDV challenge when titers of the sera elicited by them differ. Hence, we performed an immunological assay to determine the expression levels of cytokines in the sera raised by KLH-NJ85 and KLH-NJ85Δ. Four cytokines (IL 2, IFN γ, IL 6, and IL 10) were detected by ELISA. Specific pathogen free rabbit serum was used as a negative control. Except for IL 6, expression levels of IL 2, IFN γ, and IL 10 in sera raised in response to challenges by both KLH-NJ85 and KLH-NJ85Δ were higher than the negative control (**Figure S12**). It was known that high levels of IL 2 proliferate activated T cells and high levels of IFN γ activate macrophages, neutrophils, and NK cells, and then promote cell-mediated immunity for antiviral effects [32]. Also, high levels of IL10 promote B-cell proliferation and antibody responses [32]. As a result, though NJ85Δ and NJ85 lead to different titers of antibodies, both are able to stimulate similar levels of cytokine expression and activate a cell-immune response that allows rabbits to resist challenges from lethal doses of RHDV.

### Concluding remarks

In this study, we used cryoEM methods to reconstruct the structure of the RHDV capsid to an overall estimated resolution limit of 6.5 Å (5.5 Å in the shell domain) and solved the crystal structures of the S and P domains of the RHDV VP60 protein both at 2.0 Å resolution. A model of the NTA domain of VP60 was built based on the near atomic resolution cryoEM map of the RHDV inner shell. A complete pseudo-atomic model of the RHDV capsid was then built by docking all the domain structures into the cryoEM map followed by MDFF refinement [21]. Structural comparison revealed a specific P2 sub-domain of RHDV in which RHDV isolates differ most and this variation contributes to HBGA binding specificity. The most exposed surface loop, L1 (a.a. 300–318), which exhibits high sequence variation among isolates, was probed to test its ability to interact with host tissue cells and to stimulate neutralizing antibodies. Cell- and animal-based experiments with synthetic peptides derived from this loop provided strong evidence that the loop is involved in virus-host interactions and stimulates production of high-titer antibody that can protect rabbits from RHDV infection.

## Materials and Methods

### Ethics statement

Animal experiments were approved by the Harbin Veterinary Research Institute of the Chinese Academy of Agricultural Sciences. All procedures were conducted in accordance with animal ethics guidelines and approved protocols. The Animal Ethics Committee approval number was Heilongjiang-SYXK 2006-032.

### Virus purification

RHDV (HYD isolate strain) was prepared from the livers of infected rabbits. These were cut into small pieces (∼5×5×5 mm^3^) and homogenized with a glass pestle in PBS buffer (8 mM Na_2_HPO4, 1.5 mM KH_2_PO4, 2.7 mM KCl, 137 mM NaCl, pH7.4) kept between 0 and 4°C. Tissue suspensions were centrifuged for 20 min at 4,000 g. An equal volume of chloroform was added to the supernatant and the mixture was shaken vigorously by hand for 15 seconds, followed by incubation for 2∼3 min at 4°C and centrifugation at 12,000 g for 15 min at 4°C. The chloroform phase was discarded and the above steps (shaking, incubation, and centrifugation) were repeated four times. The aqueous phase was then filtered through a 0.22 µm pore-size filter and overlaid into a discontinuous sucrose gradient (30%, 40%, 50%, 60%). The gradient with the clarified liver homogenate was centrifuged at 350,000 g for 80 min at 18°C in a Beckman L8-80M centrifuge with a 75 Ti rotor. Precipitant was collected and dissolved in PBS and the final sample for cryoEM studies was purified through a 25% sucrose cushion by ultracentrifugation at 145,000 g for 3 hr (Rotor Ti-75, Beckman). The resulting pellet was resuspended in TNE buffer (50 mM Tris, 50 mM NaCl, 5 mM EDTA) and fast frozen in liquid nitrogen for storage before it was used for cryoEM studies.

### CryoEM and 3D reconstruction of the RHDV virion

Small aliquots (∼3.5 µL) of purified RHDV samples were applied to holey grids (GiG) and blotted for 3 sec in a chamber at 100% humidity using an FEI Vitrobot Mark IV and then quick plunged into liquid ethane cooled by liquid nitrogen. Images were recorded with a Gatan UltraScan4000 (model 895) 16-megapixel CCD in an FEI Titan Krios cryo-electron microscope operated at 300 keV, at a calibrated magnification of 160770 (corresponds to a pixel size of 0.933 Å at the specimen), and an electron dose of ∼20 e/Å^2^ for each micrograph. A total of 1,100 cryoEM micrographs of RHDV were recorded. The defocus and astigmatism of each micrograph were estimated with CTFFIND3 [33] and corrected using the “applyctf” routine of EMAN [34]. Image processing and 3D reconstruction were performed using EMAN [34], with Spider [35], [36] scripts embedded for correspondence analysis (CORAN) of each image class, which was wrapped in the Appion package [37]. The 3D reconstruction was computed from ∼36,000 individually boxed virus particle images. The final reconstructed density map was further sharpened by application of an amplitude correction algorithm in the program BFACTOR [38] with a negative B-factor 1/(300 Å^2^). CryoEM maps were segmented, displayed, and fitted with atomic models using UCSF Chimera [39]. All illustrations of structures were rendered using either UCSF Chimera or PyMol [40].

### Expression, purification, and crystallization of P and S domain of RHDV VP60

The fragment (a.a.228–579) covering the entire P domain of the RHDV VP60 protein was cloned into pFastEL-3G vector (from Dr. Fei Sun's lab). This construct, fused with a GST tag and a precision protease digestion site at the N-terminus, was expressed in Sf21 insect cells. After GST-column (GE Healthcare) affinity purification, Prescission Protease (GE Healthcare) digestion, anion exchange by Resource Q (GE Healthcare), and gel filtration by Superdex 75 (GE Healthcare) on a BioLogic DuoFlow system (Bio-Rad), the recombinant protein was isolated at high purity (>98%). The purified sample was buffered at pH 8.0 in 50 mM Tris-HCl, 150 mM NaCl and concentrated to 3.0 mg/ml for crystallization. We used the hanging drop, vapor diffusion method to obtain brick-shaped crystals of the P domain at 289 K in the presence of 0.1 M sodium acetate, 1.1 M succinic acid, pH 5.5 and 1.0% PEG2000MME.

The contiguous NTA and S domains (a.a. 1–230) genes of the RHDV VP60 protein were cloned into the pEXS-DH vector [41] and expressed with an N-terminal 8×His tag in *E.coli* (BL21). This construct was purified using a Ni-NTA affinity column (GE Healthcare), anion exchange chromatography using a Resource Q column (GE Healthcare), and gel filtration using a Superdex 75 column (GE Healthcare) on a BioLogic DuoFlow system (Bio-Rad). The purified protein was buffered at pH 8.0 in 50 mM Tris-HCl and concentrated to 5.0 mg/ml for crystallization. Brick-shaped crystals were obtained via hanging drop, vapor diffusion at 289 K in the presence of 0.2 M MgCl_2_, 0.1 M HEPES-Na, pH 7.0 and 30% PEG400.

### Diffraction data collection, processing and structure determination

X-ray diffraction data sets of the crystals of the P and S domains were collected to 2.0 Å at the beam line BL17U (Shanghai Synchrotron Radiation Facility, SSRF) and the beamline BL17A (Photo Factory, Japan), respectively. All diffraction data were processed and scaled using HKL2000 [42]. Two copies of the S domain constitute each asymmetric unit of the crystal with a solvent content of 34.7%. The crystal structure of the S domain was solved by molecular replacement with PHASER [43] using the VP60 S domain structure from SMSV (PDB code: 2GH8) as the initial phasing model. The structure of the RHDV S domain was built manually in COOT [44] and refined using REFMAC5 [45]. The stereochemistry of the final model was evaluated by PROCHECK [46].

The determination of the P domain crystal structure was not straightforward because molecular replacement failed to yield a correct set of phases when the crystal structure of the P domain of SMSV (PDB code: 2GH8) was used as a phasing model. Instead, the cryoEM map of the RHDV virion served as a reliable initial model; the structure of SMSV P domain was fitted into the cryoEM map and modified manually by deleting the regions outside the map in COOT [44]. This EM map-based model was used as an initial model to run molecular replacement using PHASER [43]. The solution with the highest translational likelihood gain (89.33) and Z-score (3.6) was selected for further phasing. Only diffraction data up to 3.0 Å were used for phasing as this process led to a more continuous density map compared to the map that was obtained using the complete set of diffraction data. Initial phasing yielded a clear density map for P1, but not for P2. Density in both these sub-domains was gradually improved by imposing non-crystallographic symmetry (NCS) without phase extension, and further improved by changing some residues to Ser/Thr to fit the apparent density, during several rounds of refinement by REFMAC5 [45]. Subsequently, all diffraction data to 2.0 Å were used for further phasing and refinement. Automatic model building was performed by ARP/WARP [47] and 96 out of 714 total residues could be built correctly with side chains, and this guided the building of the complete model manually in COOT [44]. The final structure of the P domain was refined to 2.0 Å in REFMAC5 [45], and its stereochemistry was evaluated by PROCHECK [46] with 94.0% of the residues in most favored regions, 5.0% in allowed regions, and 0.8% in generously allowed regions.

Statistics for the data collection, processing, and structure refinement for both the P and S domains are summarized in **Table 1**.

### Molecular dynamics flexible fitting

Molecular dynamics flexible fitting (MDFF) is a computational method that employs molecular dynamics simulation to fit atomic models into cryo-EM density maps [21], [48] and has been successfully applied recently [49], [50], [51]. The initial atomic model of VP60 was obtained by combining the NTA structure derived from cryoEM density and the crystal structures of the P and S domains. Missing loops were modeled using MODELLER [52]. After rigid body docking into the cryoEM map, proteins were solvated in a box of water molecules with 150 mM NaCl in VMD [53], using 17 Å of padding in all directions. Counter ions were added to neutralize the simulated system, which was bounded by a cubic box of dimension 460 Å and contained 9,891,665 atoms. Simulations were performed with NAMD 2.9 [54], using the CHARMM27 force field with CMAP corrections [55], [56].

### Peptide synthesis

Two peptides, NJ85 (G_300_SASYSGNNATNVLQFWYA_318_) and NJ85Δ (G_300_SASYSG_306_N_311_VLQFWYA_318_), based on the VP60 sequence of RHDV NJ/China/1985 isolate strain (GeneBank accession number: AY269825) were synthesized, labeled with FITC and conjugated onto KLH, respectively, by using the commercial service from ChinaPeptides.

### Primary cell isolation and peptide binding assay

Viable fresh rabbit hepatocytes, primary splenocytes, and kidney epithelial cells RK13 (ATCC CCL-37) [57] were harvested from a healthy male, New Zealand white rabbit by using the standard collagenase perfusion technique [58] and maintained at 37°C and 5% CO_2_ in a humidified incubator.

Based on a previous protocol [59], adherent RK13 cells, hepatocytes, and splenocytes were fixed with 30% carbinol for 30 min at room temperature. The FITC conjugated peptides (FITC-NJ85 and FITC-NJ85Δ), dissolved in phosphate buffered saline (PBS) with 10% FBS (Fetal Bovine Serum) and 3% BSA (Bovine Serum Albumin), were respectively added to different cells at a final concentration 30 ug/mL and incubated for 1 hr at room temperature. Cells were washed three times with PBS containing 0.3% BSA and 0.1% Triton-X100. The interactions between the two FITC-conjugated peptides and the three types of rabbit tissue cells were imaged with an SP5 confocal microscope (Leica Microsystems, Heidelberg, Germany). Confocal stacks were combined with Image J [60] to construct the three dimensional image.

### Animals, immunizations and serological analysis

Healthy male, New Zealand white rabbits were subcutaneously immunized with 1 mg NJ85-KLH and NJ85Δ-KLH, respectively, in Freund's complete adjuvant. Further vaccinations were performed on days 14 and 21 with 1 mg of each antigen in Freund's incomplete adjuvant. Finally, rabbit sera were collected on day 28 after the initial immunization inoculation.

Antibody titres were assessed by ELISA. Briefly, RHDV virus (HYD isolate strain) (100 µl, 1 µg/ml, incubated overnight at 4°C) were used to capture antibodies in the sera (incubated for 1 h at 37°C), which were then detected with 100 µl horseradish peroxidase-conjugated goat anti-rabbit IgG (Jingmei Biotech) per well (diluted 1∶5000 in PBS containing 0.5% Tween 20 and 10% FBS), followed by 100 µl 3,3′,5,5′-Tetramethylbenzidine (TMB) Liquid Substrate (Sigma) per well for 30 min at room temperature in the dark. End-point titers were defined as the highest plasma dilution that resulted in an absorbance value (A_450_) two times higher than that of non-immune plasma with a cut-off value of 0.05. Data are presented as log_10_ values.

For Western blot experiments, RHDV viruses (HYD isolate strain) were fractionated by SDS–PAGE on a 10% gel and blotted onto Nitrocellulose Transfer Membrane (Whatman) using a semidry electro-transfer system (Amersham Biosciences). Analysis of sera was carried out by probing with anti-KLH-NJ85 and anti-KLH-NJ85Δ raised in rabbits at a dilution of 1∶1000. The reaction was detected by horseradish peroxidase-conjugated anti-mouse IgG antibody (rabbit) and visualized by enhanced chemiluminescence. The relative densities of bands were analyzed and integrated with Image J [60].

All experimental data are expressed as means ± SD and were analyzed by a *t*-test using the SPSS 10.0 statistical software. Probability values of <0.05 were considered to be statistically significant.

### Hemagglutination and its inhibition assay

Hemagglutination (HA) of RHDV in the liver homogenates was tested according to Capucci [61]. The reaction was performed at room temperature for 30 min in PBS (pH 7.4). Two-fold serial dilution of the virus was added in a 96-well, V bottom microplate with 25 µL for each well. Then, a 1% suspension of human type O red cells was added to a final volume of 50 µL. The highest dilution of virus that caused complete hemagglutination of red cells was considered as the end point (**Figure S10A**).

Hemagglutination inhibition titers of the sera were tested as described [62]. After inactivation at 56°C for 30 min, sera were diluted two-fold serially from 1∶2 to 1∶512 respectively into PBS and added together with 8 HA units of RHDV antigen (1∶2048 dilution) into a 96-well V bottom microplate with 25 µL in each well. The plate was incubated for 1 hour at room temperature. Then, 25 µL of 1% suspension of human type O red cells was added into each well and incubated for 30 min. The highest sera dilution that caused complete inhibition was considered as the end point. Specific pathogen free rabbit serum was used as a negative control.

### Virus challenge in vivo

Healthy male, New Zealand white rabbits weighing between 3.0 and 3.5 kg were divided into three groups (*n* = 5 in each group) and raised in individual ventilated cages in a bio-safety level 3 enhanced containment laboratory approved by the Chinese Ministry of Agriculture. One group was subcutaneously immunized with 1 mg KLH-NJ85 in Freund's complete adjuvant, one group with KLH-NJ85Δ and the rest with KLH as a negative control. After immunization, those rabbits in each group were challenged intranasally with 256 hemagglutination titer of RHDV [63] and continuously housed and monitored every 24 hours for investigation of survival rate.

### Accession numbers

The isolate strain used for cryoEM study and virus challenge experiments was HYD isolate strain (GeneBank accession number: JF412629). The P and S domain of RHDV VP60 was cloned from the JX/CHA/97 isolate strain (GeneBank accession number: DQ205345). The two peptides, NJ85 and NJ85Δ, were synthesized according to the VP60 sequence of RHDV from NJ/China/1985 isolate strain (GeneBank accession number: AY269825).

The coordinates of the crystal structures of the RHDV VP60 S and P domains are deposited in the Protein Data Bank (PDB) with accession numbers 4EJR and 4EGT, respectively. The cryoEM map of the RHDV virion is deposited in the Electron Microscopy Data Bank (EMDB) with accession number EMD-5410 and its corresponding pseudo-atomic model is deposited in the PDB with accession number 3J1P.

## Supporting Information

Figure S1 **CryoEM reconstruction of RHDV capsid.** (**A**) Two dimensional reference-free image classification of the raw RHDV cryoEM particles. The particles containing the complete genome with significant density inside the shell are indicated by light-blue squares and the particles containing partial genome with less density inside are indicated by red squares. (**B**) Central cross sections of the reconstructed cryoEM map of RHDV perpendicular to the 3-, 5- and 2-fold axes, respectively. (**C**) Fourier shell correlation (FSC) plot of the cryoEM reconstruction of RHDV. The estimated resolution limit for the whole virion is 6.5 Å for an FSC threshold of 0.5 and 4.8 Å for an FSC threshold of 0.143 (blue curve). The FSC curve for just the RHDV inner shell density (shown in black) indicates a resolution 5.5 Å at FSC = 0.5.
(TIF)

Figure S2 **Comparison of the S domains of three calicivirus major capsid proteins.** Sequence alignment (**A**) and superposition (**B**) of the VP60 S domains were performed for RHDV (red, this paper), rNV (green, PDB code 1IHM), SMSV (purple, PDB code 2GH8) and FCV (yellow, PDB code 3M8L), respectively.
(TIF)

Figure S3 **Crystal structure of RHDV P domain.** (**A**) The high quality of the electron density map from the crystal structure of the RHDV VP60 P domain is contoured at 1.0σ and fitted with the coordinates. (**B**) The structure of dimeric P domains of RHDV in an asymmetric unit of the crystal is shown in ribbon form and their P1 and P2 sub-domains are colored green and pink, respectively.
(TIF)

Figure S4 **Multiple sequence alignment of P domains from different caliciviruses.** The sequences correspond to the P domains of VP60 from RHDV (this paper), rNV (PDB code 1IHM), SMSV (PDB code 2GH8) and FCV (PDB code 3M8L), respectively. The secondary structure elements of the RHDV VP60 P domain in line with **Figure 2C and D** are shown on the top row.
(TIF)

Figure S5 **Model fitting into the cryoEM map.** (**A**) **and** (**B**) The cryoEM map of RHDV capsid is fitted with its atomic model and viewed from the inside surface. The quasi-equivalent VP60 monomers, A, B and C, are colored red, yellow and blue, respectively. The A/B dimer in (**A**) and C/C dimer in (**B**) are highlighted with dashed orange polygons. The connection site between the S domain and NTA segment as well as the interaction interface within the dimer are highlighted by dashed black ellipses. (**C**) **and** (**D**) Side views of the atomic model fitted cryoEM maps of A/B and C/C capsomer (see also **Figure 3C and D**). (**E**) **and** (**F**) Model-fitting before (**E**) and after (**F**) MDFF refinement for the S domains in the region near the 3-fold axis as viewed from inside the capsid.
(TIF)

Figure S6 **Structural comparison of two models of RHDV VP60.** The model derived during the present study is compared to the model (colored magenta) in Ref. 16 (PDB code: 3ZUE) for the A (red), B (yellow), and C (blue) monomers. Two significant differences between the models are highlighted by orange dashed circles for the P2 sub-domains and by cyan dashed rectangles for the NTA regions.
(TIF)

Figure S7 **Mapping sequence variation onto the capsomer surface.** The level of conservation from the multiple sequence alignments shown in **Figure 5A** are mapped onto the surface of the RHDV capsomer, which is shown in front (left) and side (right) views. The color scheme is the same as that used in **Figure 5**.
(TIF)

Figure S8 **Flexibility of L1 loop and its high variability across RHDV strains.** (**A**) Distribution of the temperature factor (B-factor) of main chain atoms on the crystal structure of the dimeric P domains. The highest and lowest B-factors are colored red and blue, respectively. The highly flexible L1 loops are identified by black boxes. (**B**) Crystal packing of the dimeric P domains (colored yellow and red, respectively) showing that the L1 loop is not exposed to solvent, and thereby its high flexibility is not attributed to crystal packing. (**C**) Full sequence alignment of VP60 proteins from different RHDV strains JX/CHA/97, NJ85, HYD, CD/China, AST/89, Mexico89, Italy-90, France95-10, Frankurt5 and Iowa2000 and Gene Bank accession numbers ABA46865, AAP15339, AEB26305, AAS13690, CAA89265, AAG16239, ABV56612, CAD59249, ABU90735, and AAF69514, respectively. The sequences are color-coded yellow (100%), magenta (>80%), red (>60%), and white (<60%) according to sequence similarity. The black box encircles the most variable region of sequence (304–315) among the RHDV strains. The sequence alignment was performed and drawn by using Geneious (www.geneious.com).
(TIF)

Figure S9 **Quantitative plots of the anti-RHDV efficiencies of the antibodies.** (**A**) Quantitative plot of the band intensity of RHDV VP60 recognized by antibodies anti-NJ85-KLH and anti-NJ85Δ-KLH, respectively in **Figure 7C**. (**B**) The titers of the antibodies against RHDV as measured by the ELISA assay. Error bars represent the standard deviation from five independent experiments.
(TIF)

Figure S10 **Hemagglutination and its inhibition assay.** (**A**) Hemagglutination test of RHDV antigen. The virus was two-fold serial diluted from the 1^st^ well to the 21^st^ well. The highest dilution of virus that caused complete hemagglutination of red cells appeared at the 14^th^ well. The HA titer of RHDV antigen was 1∶2^14^ (1∶16384). (**B**) Hemagglutination inhibition (HI) tests for the sera raised by peptides NJ85 and NJ85Δ. HI titers of the sera were detected by using 8 hemagglutination-units of RHDV antigen (1∶2048 dilution). The sera dilutions ranged from 1∶2 to 1∶512. HI titer of sera raised by peptide NJ 85 was considered as 1∶64 and that of sera immunized by NJ85Δ as 1∶32. The SPF rabbit serum was used as a negative control and has no HI titer.
(TIF)

Figure S11 **Structural comparisons of P2 sub-domains of RHDV and MNV-1.** Both (**A**) the closed and (**B**) open (PDB code: 3LQ6) conformations of the MNV-1 P2 sub-domain [31] are used to make the comparisons. The corresponding loops (A′-B′ and E′-F′) in MNV-1 and loops (L1 and L5) in RHDV are indicated and labeled accordingly.
(TIF)

Figure S12 **Detection of cytokines in the sera.** Expression levels of IL 10, IL 6, IL 2 and IFN γ in the sera were detected using ELISA kits. The absorbance values were determined at 450 nm. The expression levels of IL 2, IFN γ, and IL 10 from the sera raised by peptides NJ85Δ and NJ85 were higher than the negative control (SPF rabbit serum) (P<0.05). All error bars represent the standard deviation from three independent experiments.
(TIF)

Movie S1 **Architecture of RHDV.** First, the model of RHDV is shown in ribbon representation and rotated. The P1 and P2 subdomains, the S domain, and the NTA domain are colored red, yellow, blue, and green, respectively. Second, the asymmetric unit (containing three VP60 monomers, A, B and C) of the RHDV capsid is picked out and the movie zoomed in this unit and shows it in different views. Third, the VP60 monomers A (red), B (yellow), and C (blue) are compared to show that the conformational differences among them result from relative movements between their P and S domains. Finally, it is shown that the S domains of the A, B, and C monomers form the closed inner shell of the RHDV capsid, and the P domains form the protruding capsomers.
(FLV)

Table S1 **Local cross-correlation coefficient (LCCC) between atomic model and cryoEM map and root mean square deviation (RMSD) between initial model and final MDFF-refined model.**
(DOCX)

Table S2 **Statistics of rabbit survival in the neutralization experiment.**
(DOCX)

Table S3 **Statistics of survivals of virus challenged rabbits that were immunized with different peptides.**
(DOCX)
