# Supplementary figures and images for: Atomic Model of Rabbit Hemorrhagic Disease Virus by Cryo-Electron Microscopy and Crystallography
Source: PLoS Pathog. 2013 Jan 17;9(1):e1003132. doi: 10.1371/journal.ppat.1003132 (PMC3547835; doi:10.1371/journal.ppat.1003132)

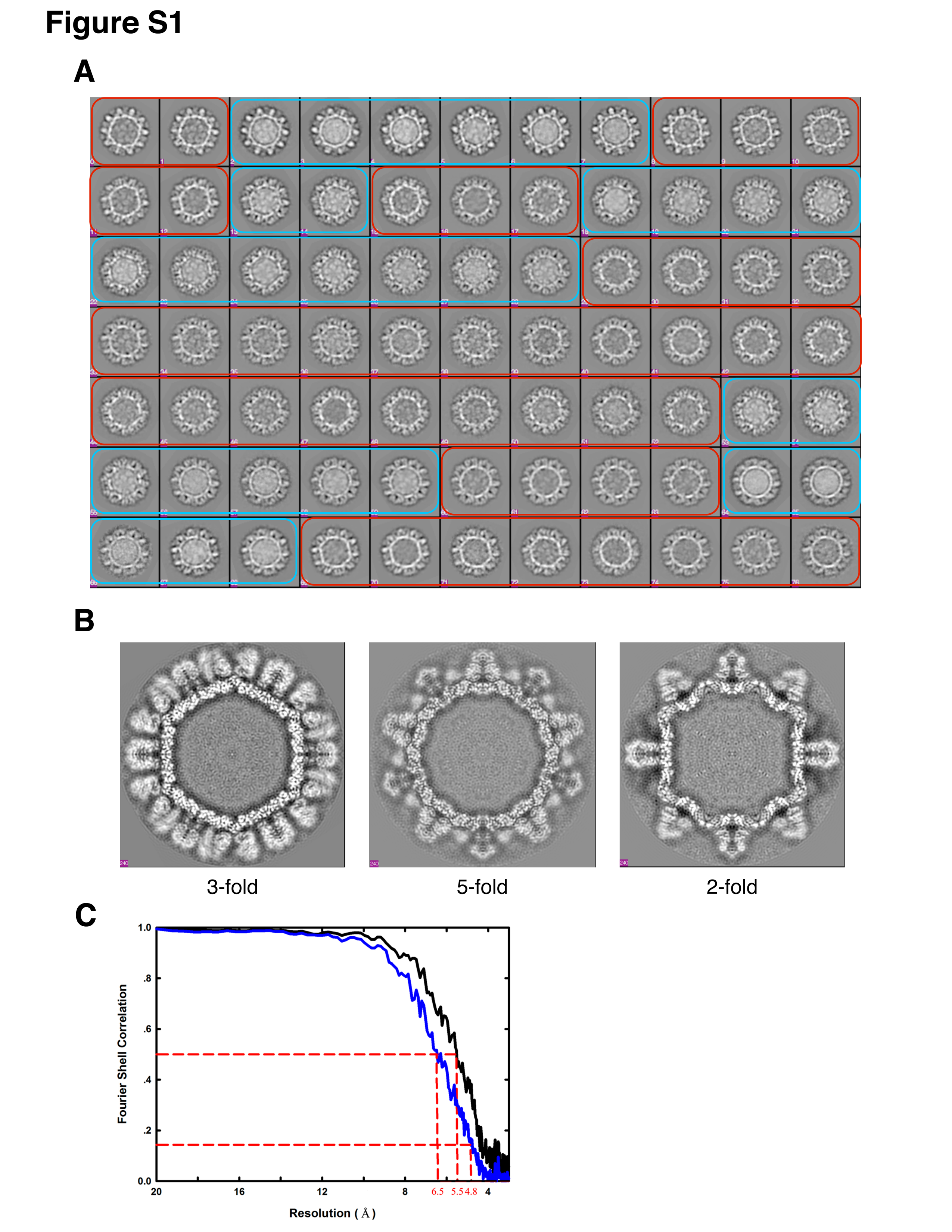

Supplement: Figure S1 — CryoEM reconstruction of RHDV capsid. (A) Two dimensional reference-free image classification of the raw RHDV cryoEM particles. The particles containing the complete genome with significant density inside the shell are indicated by light-blue squares and the particles containing partial genome with less density inside are indicated by red squares. (B) Central cross sections of the reconstructed cryoEM map of RHDV perpendicular to the 3-, 5- and 2-fold axes, respectively. (C) Fourier shell correlation (FSC) plot of the cryoEM reconstruction of RHDV. The estimated resolution limit for the whole virion is 6.5 Å for an FSC threshold of 0.5 and 4.8 Å for an FSC threshold of 0.143 (blue curve). The FSC curve for just the RHDV inner shell density (shown in black) indicates a resolution 5.5 Å at FSC = 0.5. (TIF) [file ppat.1003132.s001.tif]

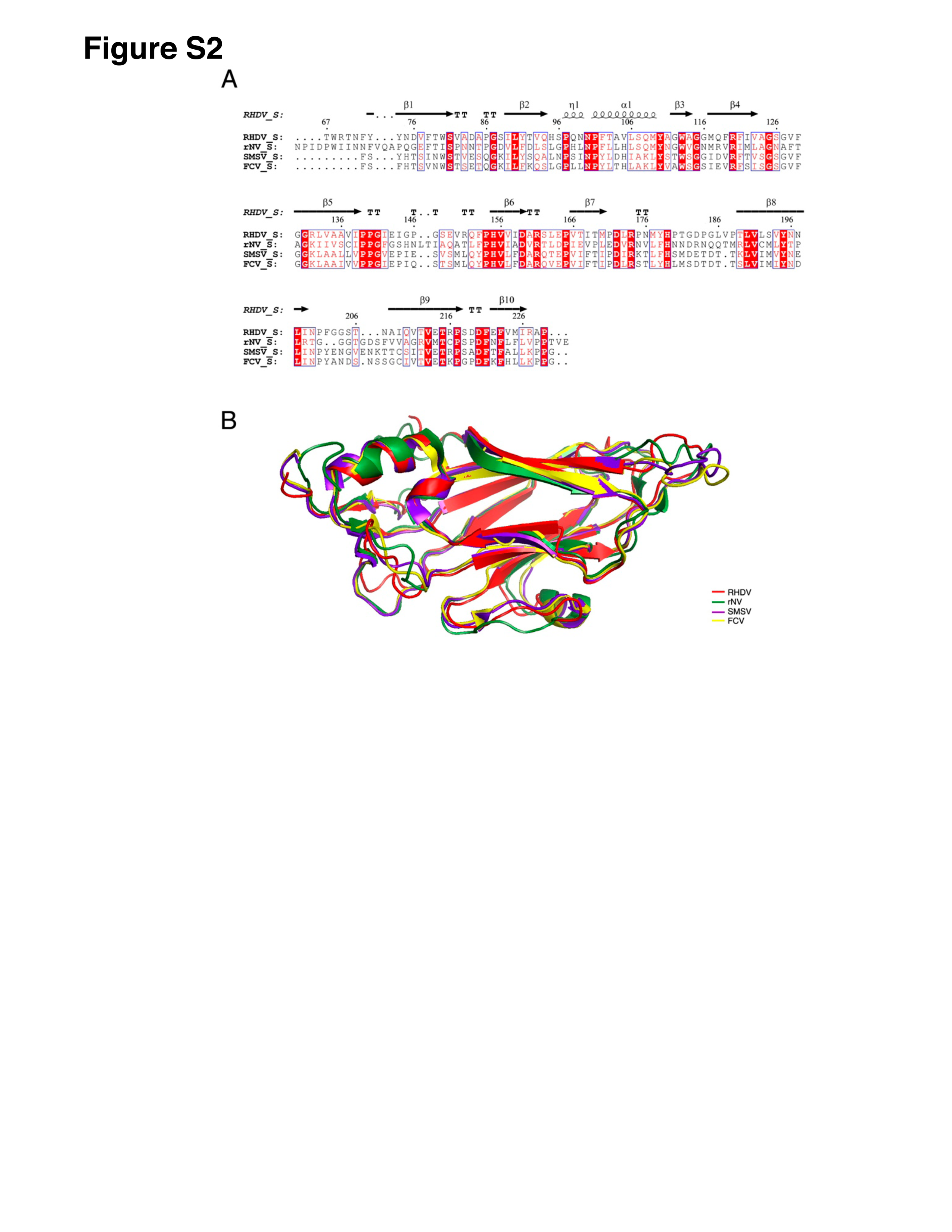

Supplement: Figure S2 — Comparison of the S domains of three calicivirus major capsid proteins. Sequence alignment (A) and superposition (B) of the VP60 S domains were performed for RHDV (red, this paper), rNV (green, PDB code 1IHM), SMSV (purple, PDB code 2GH8) and FCV (yellow, PDB code 3M8L), respectively. (TIF) [file ppat.1003132.s002.tif]

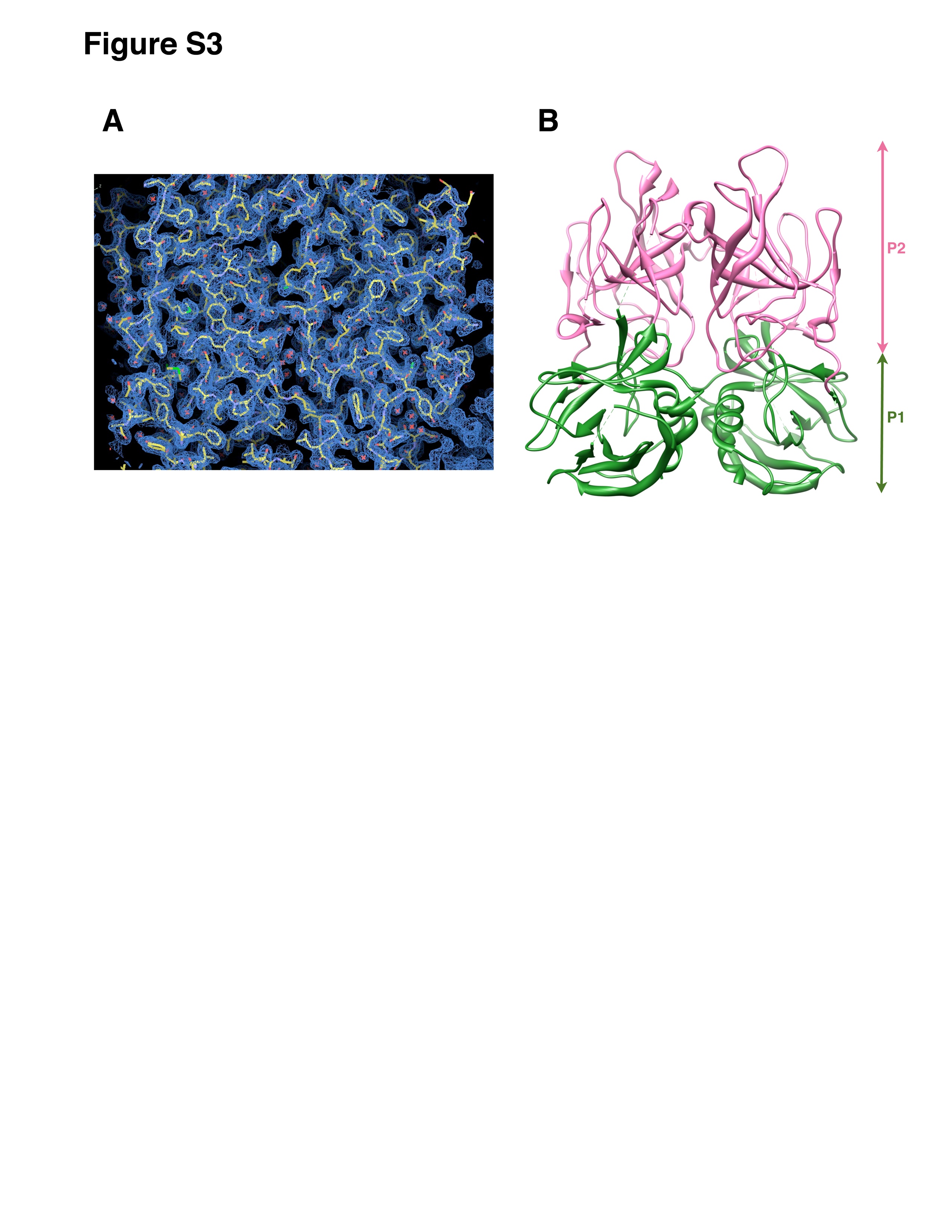

Supplement: Figure S3 — Crystal structure of RHDV P domain. (A) The high quality of the electron density map from the crystal structure of the RHDV VP60 P domain is contoured at 1.0σ and fitted with the coordinates. (B) The structure of dimeric P domains of RHDV in an asymmetric unit of the crystal is shown in ribbon form and their P1 and P2 sub-domains are colored green and pink, respectively. (TIF) [file ppat.1003132.s003.tif]

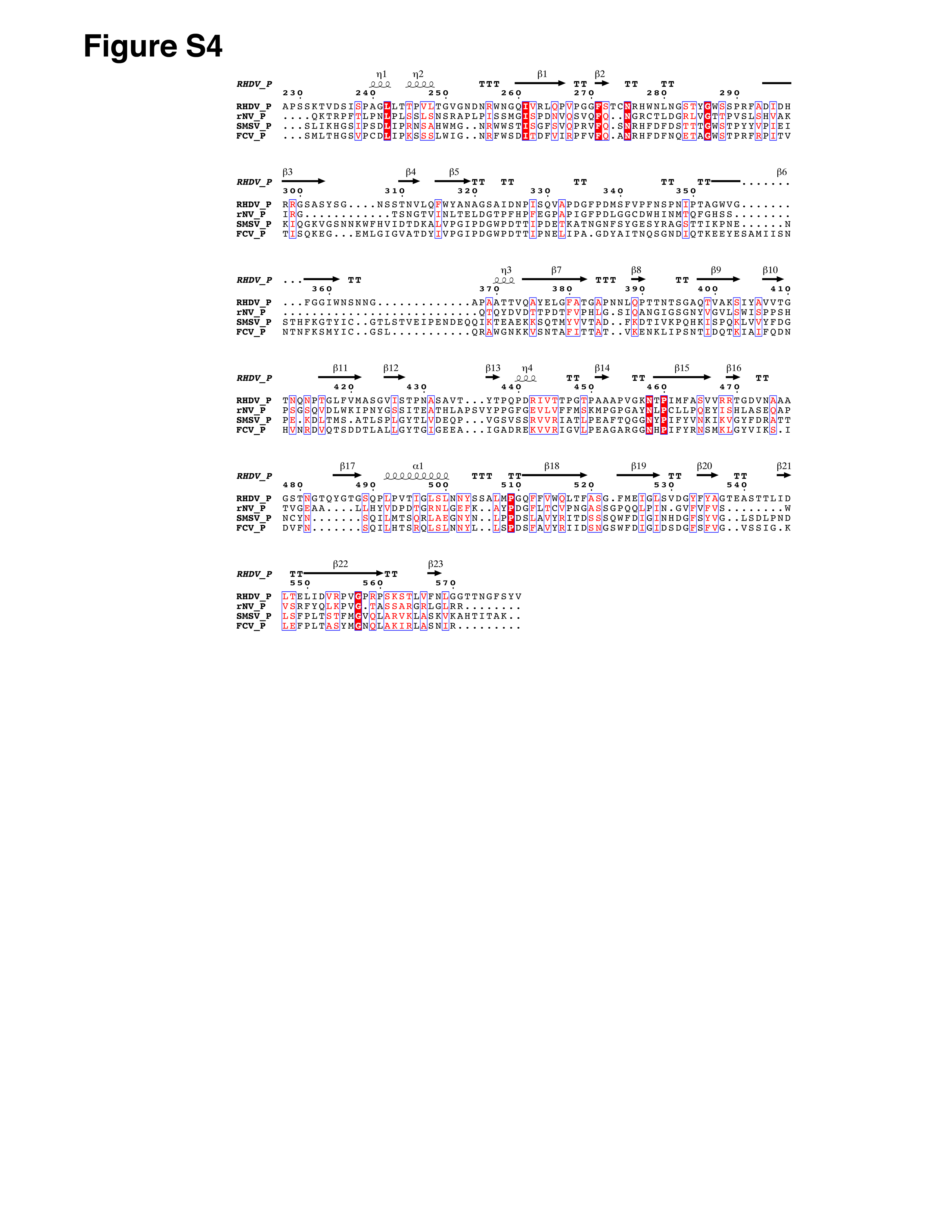

Supplement: Figure S4 — Multiple sequence alignment of P domains from different caliciviruses. The sequences correspond to the P domains of VP60 from RHDV (this paper), rNV (PDB code 1IHM), SMSV (PDB code 2GH8) and FCV (PDB code 3M8L), respectively. The secondary structure elements of the RHDV VP60 P domain in line with Figure 2C and D are shown on the top row. (TIF) [file ppat.1003132.s004.tif]

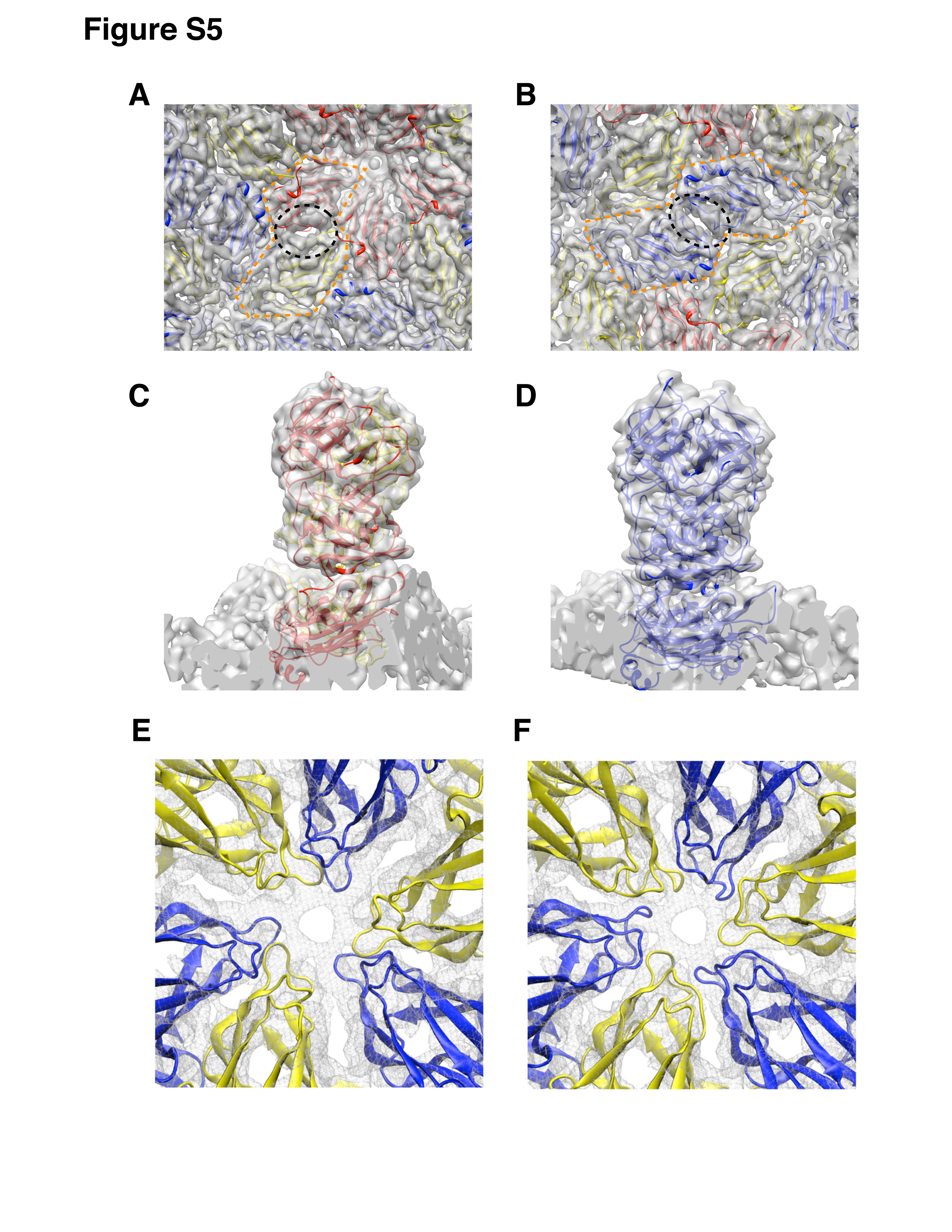

Supplement: Figure S5 — Model fitting into the cryoEM map. (A) and (B) The cryoEM map of RHDV capsid is fitted with its atomic model and viewed from the inside surface. The quasi-equivalent VP60 monomers, A, B and C, are colored red, yellow and blue, respectively. The A/B dimer in (A) and C/C dimer in (B) are highlighted with dashed orange polygons. The connection site between the S domain and NTA segment as well as the interaction interface within the dimer are highlighted by dashed black ellipses. (C) and (D) Side views of the atomic model fitted cryoEM maps of A/B and C/C capsomer (see also Figure 3C and D ). (E) and (F) Model-fitting before (E) and after (F) MDFF refinement for the S domains in the region near the 3-fold axis as viewed from inside the capsid. (TIF) [file ppat.1003132.s005.tif]

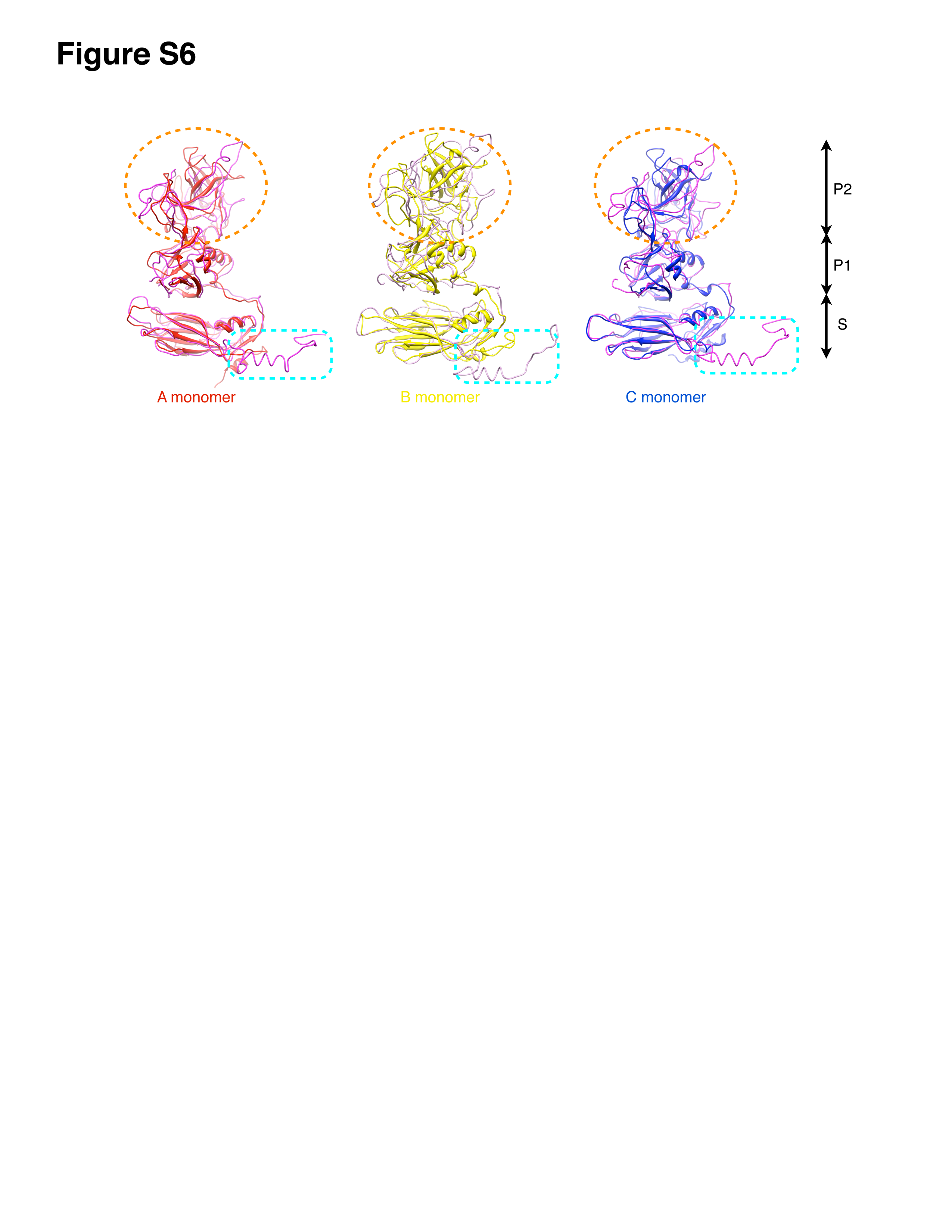

Supplement: Figure S6 — Structural comparison of two models of RHDV VP60. The model derived during the present study is compared to the model (colored magenta) in Ref. 16 (PDB code: 3ZUE) for the A (red), B (yellow), and C (blue) monomers. Two significant differences between the models are highlighted by orange dashed circles for the P2 sub-domains and by cyan dashed rectangles for the NTA regions. (TIF) [file ppat.1003132.s006.tif]

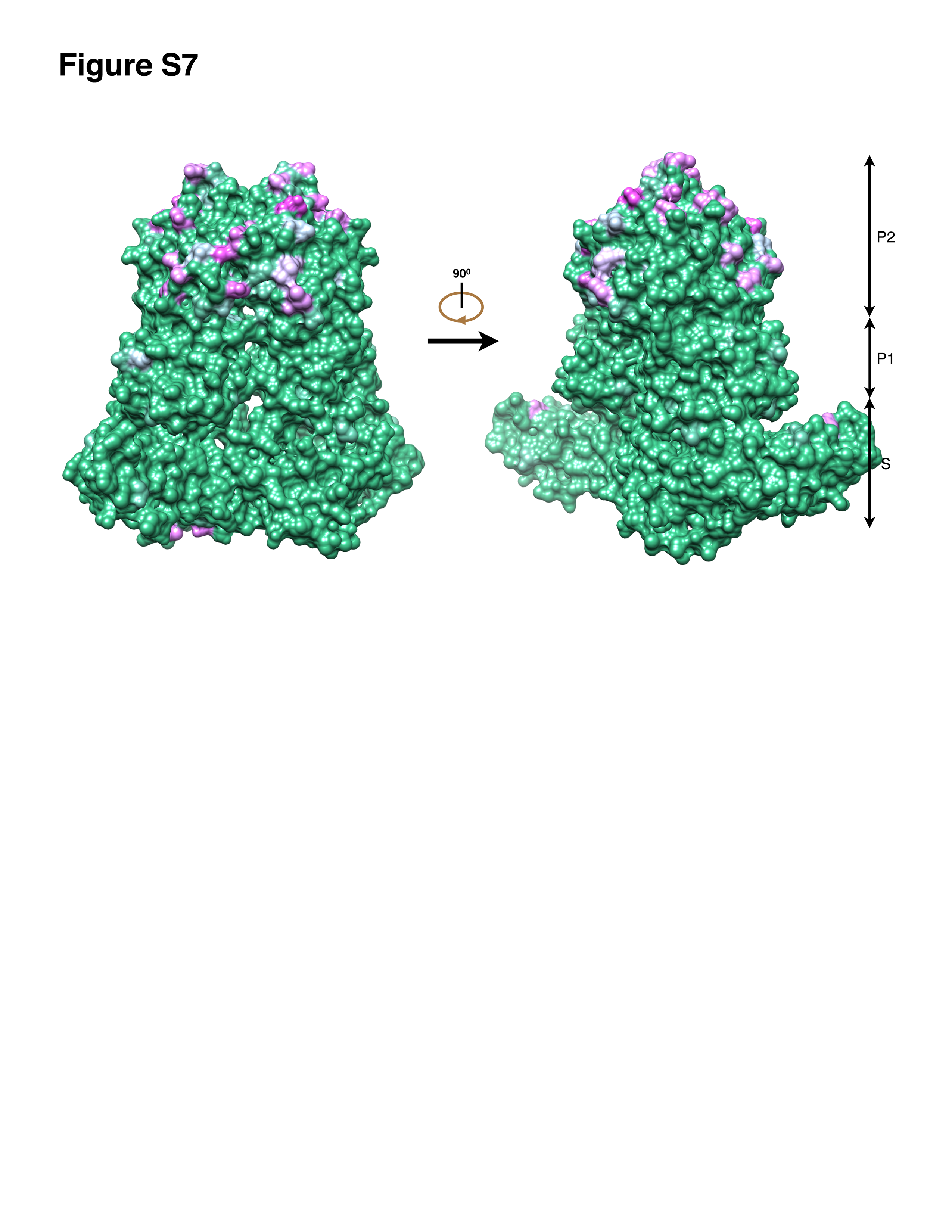

Supplement: Figure S7 — Mapping sequence variation onto the capsomer surface. The level of conservation from the multiple sequence alignments shown in Figure 5A are mapped onto the surface of the RHDV capsomer, which is shown in front (left) and side (right) views. The color scheme is the same as that used in Figure 5 . (TIF) [file ppat.1003132.s007.tif]

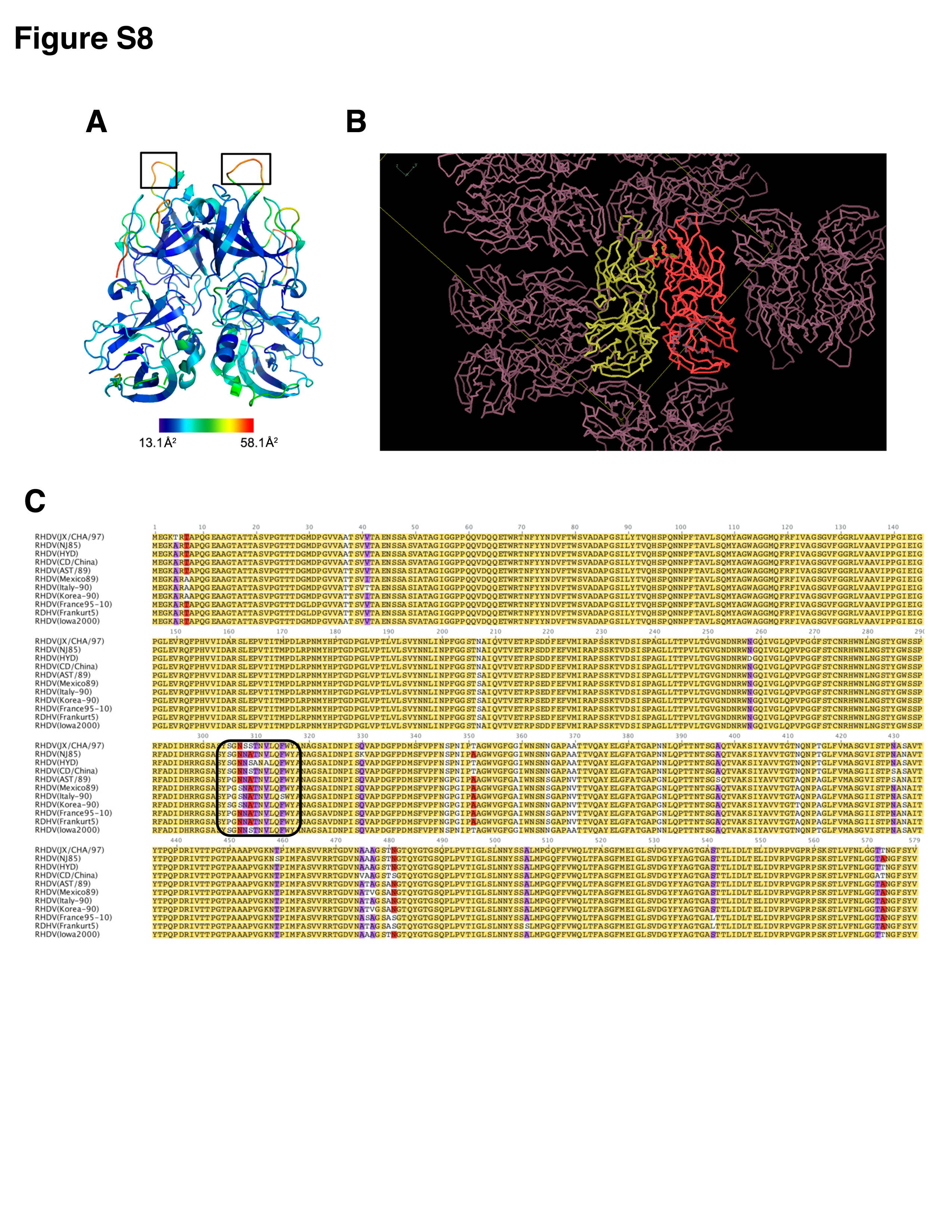

Supplement: Figure S8 — Flexibility of L1 loop and its high variability across RHDV strains. (A) Distribution of the temperature factor (B-factor) of main chain atoms on the crystal structure of the dimeric P domains. The highest and lowest B-factors are colored red and blue, respectively. The highly flexible L1 loops are identified by black boxes. (B) Crystal packing of the dimeric P domains (colored yellow and red, respectively) showing that the L1 loop is not exposed to solvent, and thereby its high flexibility is not attributed to crystal packing. (C) Full sequence alignment of VP60 proteins from different RHDV strains JX/CHA/97, NJ85, HYD, CD/China, AST/89, Mexico89, Italy-90, France95-10, Frankurt5 and Iowa2000 and Gene Bank accession numbers ABA46865, AAP15339, AEB26305, AAS13690, CAA89265, AAG16239, ABV56612, CAD59249, ABU90735, and AAF69514, respectively. The sequences are color-coded yellow (100%), magenta (>80%), red (>60%), and white (<60%) according to sequence similarity. The black box encircles the most variable region of sequence (304–315) among the RHDV strains. The sequence alignment was performed and drawn by using Geneious (www.geneious.com). (TIF) [file ppat.1003132.s008.tif]

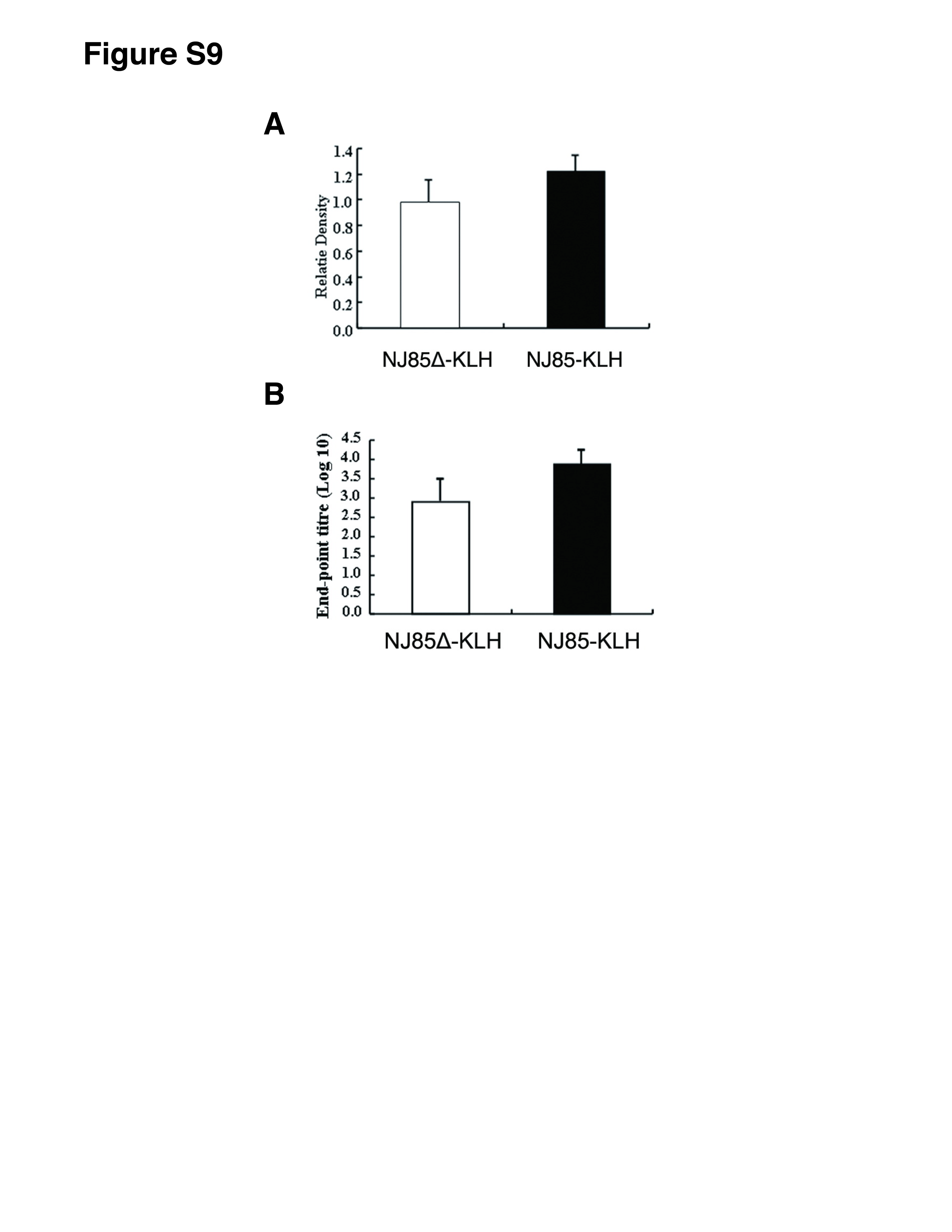

Supplement: Figure S9 — Quantitative plots of the anti-RHDV efficiencies of the antibodies. (A) Quantitative plot of the band intensity of RHDV VP60 recognized by antibodies anti-NJ85-KLH and anti-NJ85Δ-KLH, respectively in Figure 7C. (B) The titers of the antibodies against RHDV as measured by the ELISA assay. Error bars represent the standard deviation from five independent experiments. (TIF) [file ppat.1003132.s009.tif]

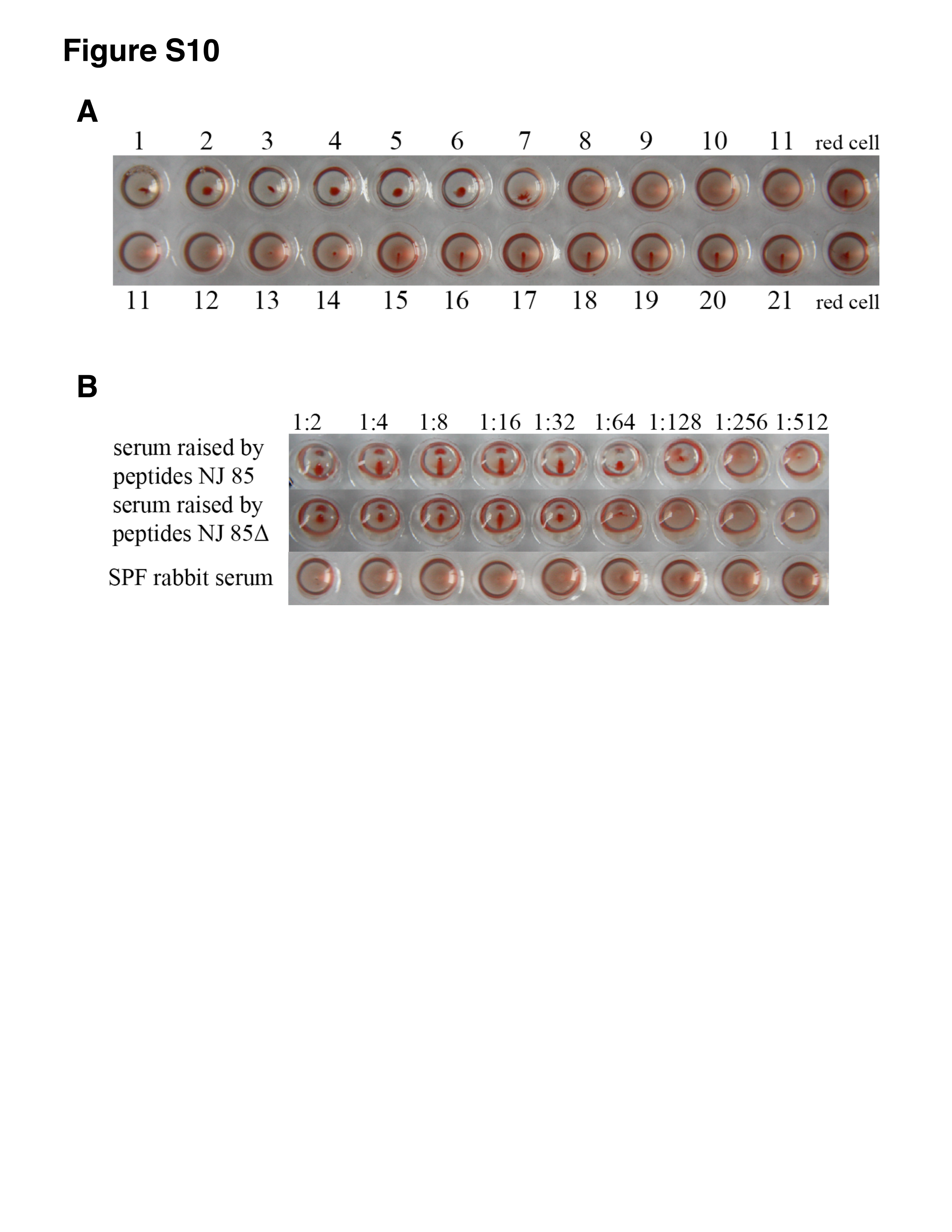

Supplement: Figure S10 — Hemagglutination and its inhibition assay. (A) Hemagglutination test of RHDV antigen. The virus was two-fold serial diluted from the 1st well to the 21st well. The highest dilution of virus that caused complete hemagglutination of red cells appeared at the 14th well. The HA titer of RHDV antigen was 1∶214 (1∶16384). (B) Hemagglutination inhibition (HI) tests for the sera raised by peptides NJ85 and NJ85Δ. HI titers of the sera were detected by using 8 hemagglutination-units of RHDV antigen (1∶2048 dilution). The sera dilutions ranged from 1∶2 to 1∶512. HI titer of sera raised by peptide NJ 85 was considered as 1∶64 and that of sera immunized by NJ85Δ as 1∶32. The SPF rabbit serum was used as a negative control and has no HI titer. (TIF) [file ppat.1003132.s010.tif]

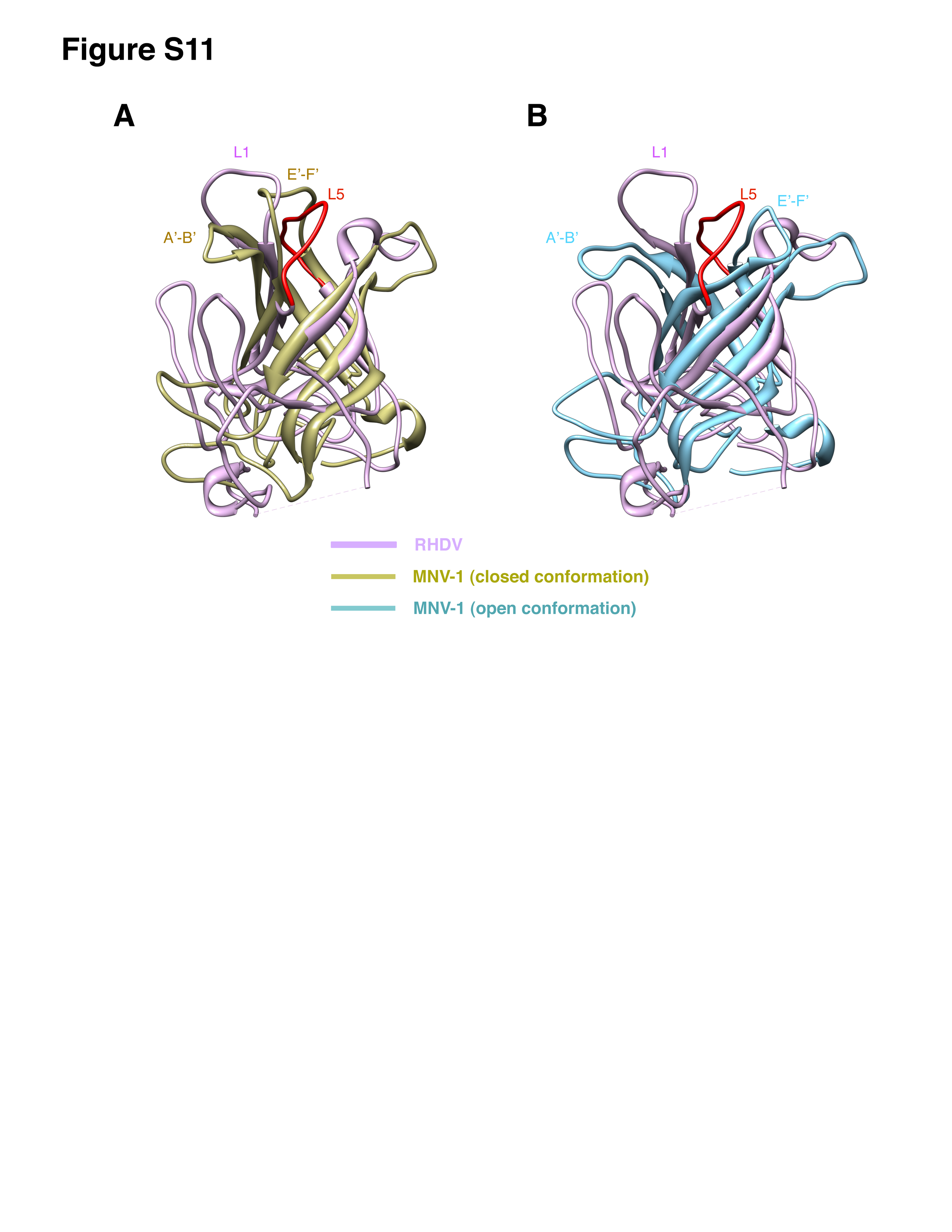

Supplement: Figure S11 — Structural comparisons of P2 sub-domains of RHDV and MNV-1. Both (A) the closed and (B) open (PDB code: 3LQ6) conformations of the MNV-1 P2 sub-domain [31] are used to make the comparisons. The corresponding loops (A′-B′ and E′-F′) in MNV-1 and loops (L1 and L5) in RHDV are indicated and labeled accordingly. (TIF) [file ppat.1003132.s011.tif]

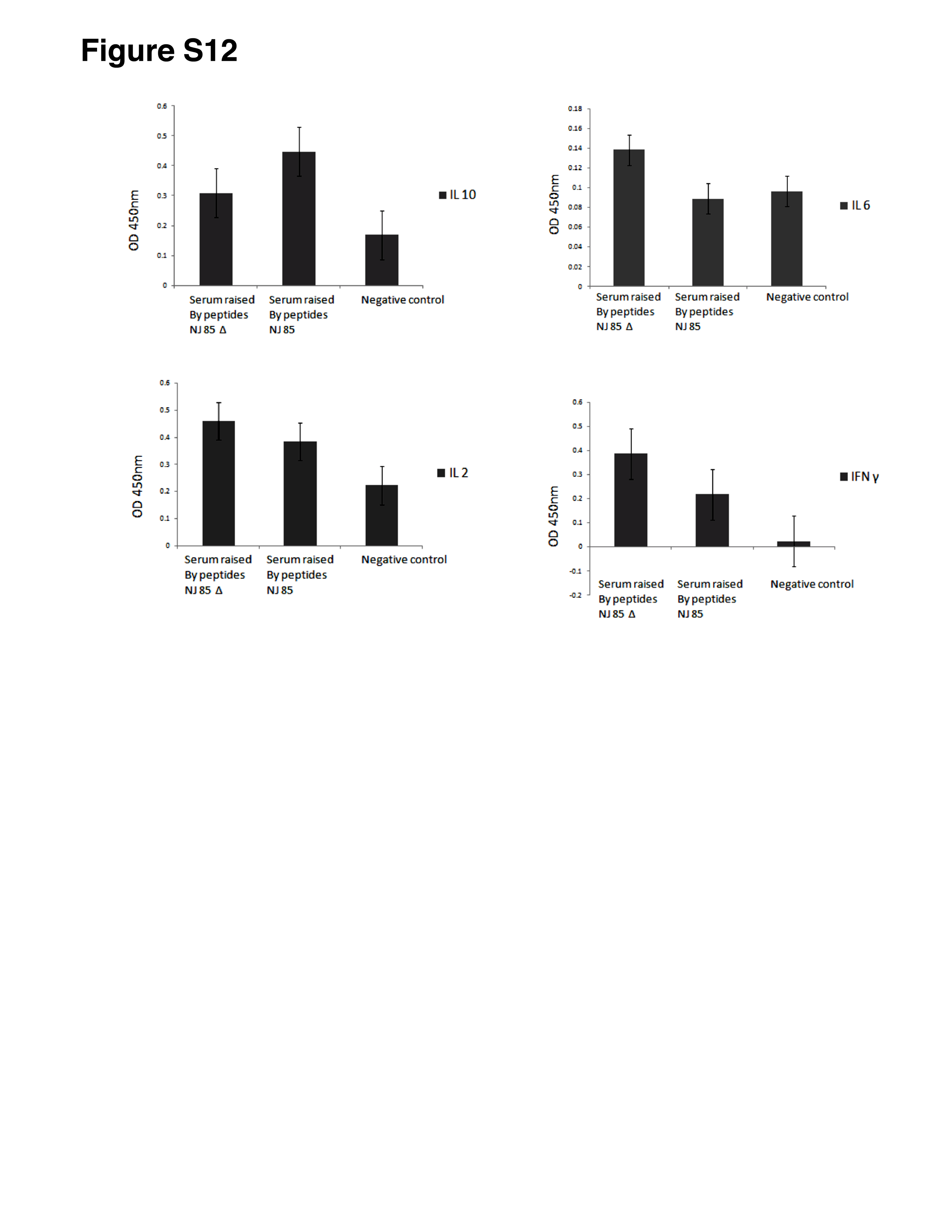

Supplement: Figure S12 — Detection of cytokines in the sera. Expression levels of IL 10, IL 6, IL 2 and IFN γ in the sera were detected using ELISA kits. The absorbance values were determined at 450 nm. The expression levels of IL 2, IFN γ, and IL 10 from the sera raised by peptides NJ85Δ and NJ85 were higher than the negative control (SPF rabbit serum) (P<0.05). All error bars represent the standard deviation from three independent experiments. (TIF) [file ppat.1003132.s012.tif]
