# Supplementary material for: Atomic Model of Rabbit Hemorrhagic Disease Virus by Cryo-Electron Microscopy and Crystallography
Source: PLoS Pathog. 2013 Jan 17;9(1):e1003132. doi: 10.1371/journal.ppat.1003132 (PMC3547835; doi:10.1371/journal.ppat.1003132)
Supplement: Table S1 — Local cross-correlation coefficient (LCCC) between atomic model and cryoEM map and root mean square deviation (RMSD) between initial model and final MDFF-refined model. (DOCX) [file ppat.1003132.s014.docx]

**Table S1. Local cross-correlation coefficient (LCCC) between atomic model and cryoEM map and root mean square deviation (RMSD) between initial model and final MDFF-refined model.**

| **Domain** | **LCCC^##^** | | **RMSD (Å)** |
| --- | --- | --- | --- |
|  | Initial model | MDFF-refined model |  |
| Whole capsid | 0.473 | 0.634 | 2.445 |
| S domain | 0.452 | 0.673 | 2.503 |
| P domain | 0.567 | 0.661 | 2.107 |
| P1 subdomain | 0.489 | 0.554 | 1.980 |
| P2 subdomain | 0.523 | 0.614 | 2.208 |
| Subunit A | 0.563 | 0.669 | 2.403 |
| Subunit B | 0.479 | 0.613 | 2.594 |
| Subunit C | 0.517 | 0.663 | 2.332 |

## LCCC was computed between the simulated map of the atomic model and the corresponding cryoEM map. Each map was set with the threshold of 0.5 σ above the mean.
