# Supplementary material for: Atomic Model of Rabbit Hemorrhagic Disease Virus by Cryo-Electron Microscopy and Crystallography
Source: PLoS Pathog. 2013 Jan 17;9(1):e1003132. doi: 10.1371/journal.ppat.1003132 (PMC3547835; doi:10.1371/journal.ppat.1003132)
Supplement: Table S2 — Statistics of rabbit survival in the neutralization experiment. (DOCX) [file ppat.1003132.s015.docx]

**Table S2. Statistics of rabbit survival in the neutralization experiment**

| Experimental group | Group I^@^ | Group II^#^ | Group III^$^ |
| --- | --- | --- | --- |
| Number of rabbits | 5 | 5 | 5 |
| [Immune serum](app:ds:%5b%E5%85%8D%E7%96%AB%5d%20immune%20serum) | SPF (control) | NJ85-KLH | NJ85Δ-KLH |
| Number of surviving rabbits at specified time periods | | | |
| 0-24hr | 5 | 5 | 5 |
| 24-48hr | 4 | 5 | 5 |
| 48-72hr | 1 | 5 | 5 |
| 72-96hr | 0 | 5 | 5 |
| <10 days | 0 | 5 | 5 |

^@^ Group I is the negative control with the serum (1:32 diluted) of specific pathogen free (SPF) rabbit mixed with 256 hemagglutination units of RHDV and used to infect 5 rabbits.

^#^ Group II is the neutralization assay group with the serum (1:32 diluted) raised by NJ85-KLH mixed with 256 hemagglutination units of RHDV and used to infect 5 rabbits.

^$^ Group III is the neutralization assay group with the serum (1:32 diluted) raised by NJ85Δ-KLH mixed with 256 hemagglutination units of RHDV and used to infect 5 rabbits.
