# Supplementary material for: Atomic Model of Rabbit Hemorrhagic Disease Virus by Cryo-Electron Microscopy and Crystallography
Source: PLoS Pathog. 2013 Jan 17;9(1):e1003132. doi: 10.1371/journal.ppat.1003132 (PMC3547835; doi:10.1371/journal.ppat.1003132)
Supplement: Table S3 — Statistics of survivals of virus challenged rabbits that were immunized with different peptides. (DOCX) [file ppat.1003132.s016.docx]

**Table S3. Statistics of survivals of virus challenged rabbits that were immunized with different peptides.**

| Experimental group* | | Group I | Group II | Group III |
| --- | --- | --- | --- | --- |
| Number of rabbits | | 5 | 5 | 5 |
| Immunization | | KLH (control) | NJ85-KLH | NJ85Δ-KLH |
| Number of surviving rabbits at specified time periods | | | | |
| 1^st^ Experiment | 0-24hr | 5 | 5 | 5 |
|  | 24-48hr | 5 | 5 | 5 |
|  | 48-72hr | 3 | 5 | 5 |
|  | 72-96hr | 0 | 5 | 5 |
|  | <10 days | 0 | 5 | 5 |
| 2^nd^ Experiment | 0-24hr | N.A. | 5 | 5 |
|  | 24-48hr | N.A. | 5 | 5 |
|  | 48-72hr | N.A. | 5 | 5 |
|  | 72-96hr | N.A. | 5 | 5 |
|  | <10 days | N.A. | 5 | 5 |
| 3^rd^ Experiment | 0-24hr | N.A. | 5 | 5 |
|  | 24-48hr | N.A. | 5 | 5 |
|  | 48-72hr | N.A. | 5 | 5 |
|  | 72-96hr | N.A. | 5 | 5 |
|  | <10 days | N.A. | 5 | 5 |
| 4^th^ Experiment | 0-24hr | N.A. | 5 | 5 |
|  | 24-48hr | N.A. | 5 | 5 |
|  | 48-72hr | N.A. | 5 | 5 |
|  | 72-96hr | N.A. | 5 | 5 |
|  | <10 days | N.A. | 5 | 5 |

* The virus challenge experiment for the control group was performed only once. The repeated virus challenge experiments were performed using the same amount of virus as used in the first experiment.
